# Optical Properties of Secondary Organic Aerosol Produced by Nitrate Radical Oxidation of Biogenic Volatile Organic Compounds

**DOI:** 10.1021/acs.est.0c06838

**Published:** 2021-02-17

**Authors:** Quanfu He, Sophie Tomaz, Chunlin Li, Ming Zhu, Daphne Meidan, Matthieu Riva, Alexander Laskin, Steven S. Brown, Christian George, Xinming Wang, Yinon Rudich

**Affiliations:** † Department of Earth and Planetary Sciences, Weizmann Institute of Science, Rehovot 76100, Israel; ‡ Univ Lyon, Université Claude Bernard Lyon 1, CNRS, IRCELYON, F-69626 Villeurbanne, France; § State Key Laboratory of Organic Geochemistry and Guangdong Key Laboratory of Environmental Protection and Resources Utilization, Guangzhou Institute of Geochemistry, Chinese Academy of Sciences, Guangzhou 510640, China; ∥ University of Chinese Academy of Sciences, Beijing 100049, China; ⊥ Department of Chemistry, Purdue University, West Lafayette, Indiana 47907, United States; # Chemical Sciences Division, Earth System Research Laboratory, National Oceanic and Atmospheric Administration, 325 Broadway, Boulder, Colorado 80305, United States; ▽ Department of Chemistry, University of Colorado, 216 UCB, Boulder, Colorado 80309, United States; ○ Center for Excellence in Urban Atmospheric Environment, Institute of Urban Environment, Chinese Academy of Sciences, Xiamen 361021, China

## Abstract

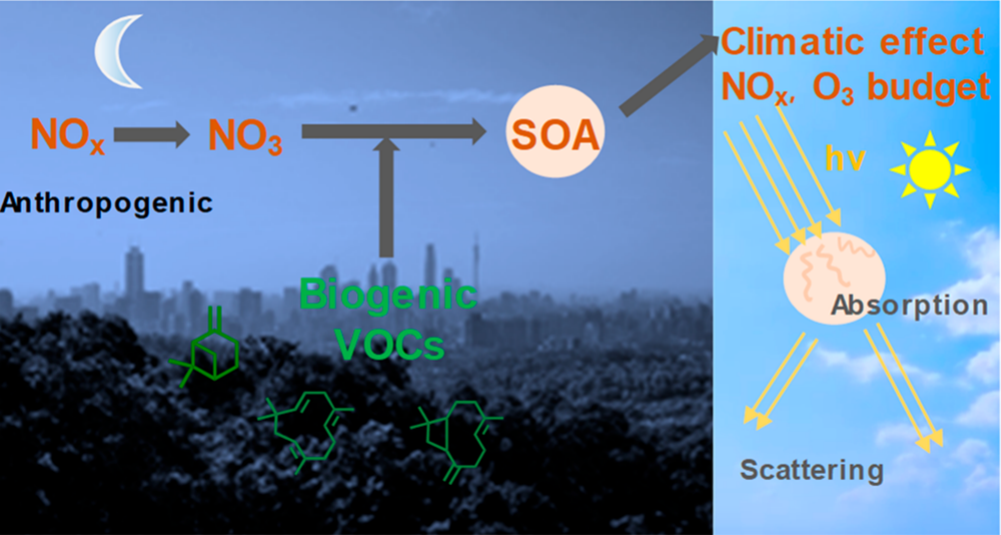

Nighttime oxidation of biogenic volatile organic compounds (BVOCs) by nitrate radicals (NO_3_·) represents one of the most important interactions between anthropogenic and natural emissions, leading to substantial secondary organic aerosol (SOA) formation. The direct climatic effect of such SOA cannot be quantified because its optical properties and atmospheric fate are poorly understood. In this study, we generated SOA from the NO_3_· oxidation of a series BVOCs including isoprene, monoterpenes, and sesquiterpenes. The SOA were subjected to comprehensive online and offline chemical composition analysis using high-resolution mass spectrometry and optical properties measurements using a novel broadband (315–650 nm) cavity-enhanced spectrometer, which covers the wavelength range needed to understand the potential contribution of the SOA to direct radiative forcing. The SOA contained a significant fraction of oxygenated organic nitrates (ONs), consisting of monomers and oligomers that are responsible for the detected light absorption in the 315–400 nm range. The SOA created from β-pinene and α-humulene was further photochemically aged in an oxidation flow reactor. The SOA has an atmospheric photochemical bleaching lifetime of >6.2 h, indicating that some of the ONs in the SOA may serve as atmosphere-stable nitrogen oxide sinks or reservoirs and will absorb and scatter incoming solar radiation during the daytime.

## 1 Introduction

Atmospheric secondary organic aerosols (SOAs) affect radiative forcing by aerosol–radiation interactions and through aerosol–cloud interactions.^1,2^ Specifically, SOAs contain light-absorbing compounds, also called brown carbon (BrC), and play a significant role in the direct climate forcing on regional and local scales.^3,4^ Owing to the high emission rates and high reactivities with primary atmospheric oxidants, such as ozone, the hydroxyl radical (OH·), and the nitrate radical (NO_3_·), vegetation-emitted biogenic volatile organic compounds (BVOCs), such as isoprene (C_5_H_8_), monoterpenes (C_10_H_16_), and sesquiterpenes (C_15_H_24_), are the major contributors to the global SOA burden.^5−9^

Whereas OH· and ozone (O_3_) play a key role during daytime atmospheric oxidation, NO_3_· is a dominant oxidant at night, especially in environments affected by anthropogenic emissions.^10^ NO_3_· is formed by the reaction of nitrogen dioxide and O_3_ and reaches atmospheric concentrations up to hundreds of parts per trillion (ppt).^11,12^ Field studies have shown that under conditions with moderate to high BVOC levels, NO_3_· predominantly reacts with BVOCs^12^ to produce multifunctional compounds such as organic nitrates (ONs).^13−16^ Because of their semivolatile/low-volatility nature, ONs can partition in the particle phase either by condensing onto pre-existing particles or by forming new SOA particles.^13,17,18^ Chamber studies have shown that the SOA mass yields from BVOC + NO_3_· reactions vary between 0.2 and 146% and that the ON molar yields range between 10 and 78%.^7^ The results from field measurements have also shown that the nocturnal NO_3_-initiated oxidation of BVOCs contributes a significant fraction to ambient particulate nitrates^19−22^ and organic aerosols^23−26^ that influence the air quality, human health, and the climate. Moreover, particle-phase ONs can either release nitrogen oxides (NO_*x*_ = NO + NO_2_) back into the atmosphere via further oxidation reactions and photolysis or act as terminal NO_*x*_ sinks through hydrolysis and particle deposition. Therefore, ONs play essential roles in the atmosphere and biosphere because they affect tropospheric O_3_ production and the global nitrogen cycle.

Although the oxidation of BVOCs by NO_3_· represents a critical interaction between anthropogenic and biogenic emissions, the direct radiative effects of the SOA from this process are not well constrained, in part because their optical properties are not yet insufficiently described.^27^ Whereas most of the existing literature on the optical properties of BVOC-derived SOAs has mainly focused on OH· oxidation or ozonolysis,^27−33^ studies on the optical properties of the biogenic SOA formed by NO_3_· oxidation (BSOA_NO_3__) are rare. The few studies that examined the BSOA_NO_3__ reached partially contradictory conclusions.^34−37^ For instance, the real part of the refractive index (RI) for the SOA from the NO_3_· oxidation of β-pinene and limonene was higher than those observed following OH- and ozone-initiated terpene oxidation.^35,37^ However, the real part of the RI for the SOA from the NO_3_· oxidation of isoprene seems to be similar to that of OH· and ozone-initiated oxidation.^34^ Moreover, absorption was not detected for the SOA from the NO_3_· oxidation of isoprene, β-pinene, and limonene, but significant light absorption at 355 and 405 nm was detected for the SOA formed by NO_3_·+ α-pinene.^36^ Washenfelder et al.^38^ measured aerosol optical properties at a forest site in rural Alabama during the 2013 Southern Oxidant and Aerosol Study (SOAS) campaign. They reported that ∼7% of BrC absorption could be attributed to the less oxidized oxygenated organic aerosol (LO-OOA) that reached a diel maximum at night and was correlated with particle-phase ONs, formed by nighttime reactions between monoterpenes and NO_3_·.^23^ These findings suggest that the SOA produced from reactions of NO_3_· with BVOCs may be a nighttime source of BrC that may affect the direct radiative effect of the SOA through the scattering and absorption of solar radiation. The optical properties of the BSOA_NO_3__ and its fate during daytime photooxidation remain unclear.

In this study, the representative BSOA_NO_3__ was produced by reactions of the most common BVOCs, such as isoprene, terpenes, and sesquiterpenes, with NO_3_·. We determined the scattering and absorption optical properties of the BSOA_NO_3__ over a very broad wavelength range (315–650 nm) for the first time. These optical properties are needed to understand their potential contributions to direct radiative forcing. We investigated the relationship between the SOA formation mechanism, the SOA’s chemical composition, and the measured optical properties. We show that the absorbing particulate organic nitrates have a lifetime >6 h upon the transition from nighttime to daytime oxidation. This study thus emphasizes the role of this important chemistry in the climate, air quality, and atmospheric nitrogen cycle.

## 2 Methods

### 2.1 SOA Generation with NO_3_· Oxidation

BVOCs (isoprene, monoterpenes (β-pinene and δ^3^-carene), and sesquiterpenes (α-cedrene, β-caryophyllene, and α-humulene)) were introduced into a glass oxidation flow reactor (OFR_NO_3__, L: 70 cm, ID: 7 cm) from a temperature-controlled glass reservoir. The target mixing ratio of the VOCs was achieved by controlling the flow rate through the glass reservoir and the bath temperature (−50 to +50 °C). The NO_3_· radical was produced by the thermal decomposition of synthetic N_2_O_5_ (Supporting Information (SI), Text S1). The initial mixing ratio of N_2_O_5_ was measured by a cavity ring-down system working at 662 nm (Text S2). Pure nitrogen that had been passed through the N_2_O_5_ crystal cold trap was mixed with dry synthetic air containing the BVOCs in the OFR_NO_3__ to produce BSOA_NO_3__ particles by homogeneous nucleation and condensation following the NO_3_· oxidation. The produced particles were then subjected to online and offline chemical–physical analysis (Figure S1). The total laminar flow in the reactor was 1.0 L min^–1^ (Reynolds number ≈ 20) with a corresponding residence time of 162 s. The initial conditions, including the BVOC mixing ratios and N_2_O_5_/VOC ratios, are summarized in Table S1.

### 2.2 Photochemical Aging and Photolysis of the BSOA_NO_3__

The BSOA_NO_3__ produced in the OFR_NO_3__ from β-pinene and α-humulene was further aged by OH· and photolysis in a potential aerosol mass (PAM) oxidation flow reactor (OFR). Gas-phase species produced in the OFR_NO_3__ were removed by a charcoal denuder before the PAM reactor. OH· was generated by UV photolysis (at 254 nm) of 19.6 ppmv O_3_ under 37.5% relativ humidity (RH). The total flow rate in the PAM was 3.2 L min^–1^, with a corresponding residence time of 252 s. The operational details can be found in our previous study.^33^ OH· exposure (the combination of OH· concentration and residence time) was determined by tracking the decay of SO_2_ in the PAM reactor. The equivalent OH· aging time was 24 h (assuming a daily average OH· concentration of 1.5 × 10^6^ molecules cm^–3^). Although the O_3_ concentration is higher than that of OH·, the much higher reactive uptake coefficient and reactivity of OH· ensure that the OH· plays a major role in the chemical aging process in the PAM reactor. Photolysis experiments were performed in the PAM reactor in the absence of O_3_ for comparison with the OH· aging experiments. Because the light emission spectrum of the UV lamps inside the PAM reactor is different from the ambient solar spectrum, the photolysis in the PAM reactor is converted to effective photolysis under ambient conditions by considering the actinic flux and the quantum yield of the SOA products. The calculation of the effective photolysis time is briefly described here with more information in the SI (Text S3, Figure S2). The quantum yields for the photolysis reactions of the SOA constitutes are unknown. In the generated SOA, many carbonyl and nitrate groups were detected. Moreover, the extracted absorption spectra (Figure S3) suggest the presence of carbonyl nitrates. (See Section 3.3.) Thus we assume a unified quantum yield of 0.9, as recommended for carbonyl nitrates by previous studies.^39,40^ The photolysis rate under the experimental conditions is then integrated over the 250–350 nm spectrum by considering the light absorption, quantum yield, and photon flux in the PAM. In addition, solar photolysis rates for the BSOA_NO_3__, considering the daily averaged actinic flux under cloudless ground-level conditions (Rehovot, Israel on December 17, 2019, albedo of 0.19), were also estimated. Dividing the photolysis effect (the combined product of the photolysis time and the photolysis rate) in the PAM by the solar photolysis rate yields the effective photolysis time, which was ∼0.8 h.

### 2.3 Chemical Box Modeling and Photolysis Time Estimation

To track the oxidation process in the OFR, a chemical-box model that includes gas-phase reactions of BVOCs + NO_3_,^41^ conversions between NO_3_· and N_2_O_5_, the heterogeneous reactive uptake of NO_3_· and N_2_O_5_,^42,43^ and wall losses of NO_3_· and N_2_O_5_ was used to investigate the fates of BVOCs, NO_3_·, and N_2_O_5_ in the OFR (Text S4, Tables S2–S4, and Figure S4). Because of the high reaction rates of BVOCs with NO_3_·, the BVOCs were completely (>99%) consumed in the OFR, except for isoprene (60%), which has a much slower rate constant with NO_3_ as compared with the other studied BVOCs. The N_2_O_5_ loss was dominated by wall loss (34–84%), whereas the thermal dissociation of N_2_O_5_ to produce NO_3_· was also significant (13–64%), as shown in Table S3. For experiments run at a N_2_O_5_/VOC ratio of <3, the amount of NO_3_· or N_2_O_5_ taken up by the particles was negligible compared with the consumption of NO_3_· by VOCs. However, at a high N_2_O_5_/VOC ratio (>3), the NO_3_· and N_2_O_5_ uptake by particles became considerable, indicating the importance of the heterogeneous reaction in the reactor. Moreover, the integrated NO_3_· exposures (NO_3_ exposure = ∑_0_^*t*^[NO_3_] d*t*) throughout the OFR ranged between 5.4 and 64.9 × 10^11^ molecules cm^–3^ s, which equals 0.3–3.5 h of ambient exposure by NO_3_·, assuming a typical concentration of 20 pptv at night.^44,45^

### 2.4 Online and Offline Chemical–Physical Characterization of the BSOA_NO_3__

The particle size distribution was continuously monitored with a scanning mobility particle sizer (SMPS, TSI) and an aerosol aerodynamic classifier (AAC, Cambustion, U.K.). The ratio of the aerodynamic and mobility size was then used to determine the particle effective density. A high-resolution time-of-flight aerosol mass spectrometer (HR-Tof-AMS, Aerodyne) was employed to measure the nonrefractory components (e.g., organics, nitrate) of the SOA in alternating V and W mode. Elemental ratios (e.g., H/C, O/C, N/C) and the fragment composition were extracted and corrected.^46,47^ The detected ions in the mass spectra were classified into five categories based on their elemental compositions, namely, hydrocarbon-like (C_*x*_H_*y*_^+^), less oxygenated (C_*x*_H_*y*_O^+^), more oxygenated (C_*x*_H_*y*_O_*z*_^+^), nitrogen-containing (C_*x*_H_*y*_O_*i*_N_*j*_^+^) organic components, and nitrogen oxides (NO_*y*_^+^), where *x*, *y*, *i*, and *j* ≥ 1 and *z* > 1.

SOA particles were collected on PTFE filters (0.45 μm porosity, 47 mm diameter, Whatman). Filters were stored at −20 °C before analysis. The filters were extracted, and the filtrate was concentrated and analyzed by ultra-high-performance liquid chromatography (UPLC) equipped with a photodiode array (PDA) detector (spectra detection range of 200–800 nm) followed by a Q-Exactive hybrid quadrupole–Orbitrap mass spectrometer (Orbitrap MS) with a standard heated electrospray ionization^48^ source. The raw data were acquired using Xcalibur (Thermal Scientific) software. The data were then processed with an open-source software toolbox, MZmine 2.39 (http://mzmine.github.io/), to perform peak deconvolution and chromatogram construction. Formula assignments were completed using the following constraints: C ≤ 50, H ≤ 100, N ≤ 4, O ≤ 50, and Na ≤ 1. (The latter is for positive mode only.) Details of the sample preparation, column separation, instrument configurations, and settings of MZmine 2.39 are provided in Text S5.

### 2.5 Optical Properties Measurement and RI Retrieval

The light extinction by size-selected SOA particles in the solar spectral region (315–650 nm) was measured by a two-channel broadband cavity-enhanced spectrometer (BBCES). The UV channel measures the light extinction between 315 and 350 nm (BBCES_UV_),^49,50^ and the visible channel works between 380 and 650 nm (BBCES_vis_).^33^ High-reflectivity mirrors (FiveNine Optics, U.S.) were installed in the BBCES_vis_. The mirror loss measured using N_2_ and He ranged from 86 to 494 ppm in the wavelength range of 380–650 nm. The low mirror loss ensures high sensitivity and low uncertainty in the aerosol light extinction measurements. The complex refractive index (RI = *n* + *ik*) is an intrinsic optical property of a particle. The real (*n*) and imaginary (*k*) parts of the complex RI are indicative of scattering and absorption, respectively. The complex RI of the aerosols was retrieved by extinction measurements of several particle sizes (175 to 325 nm with 25 nm steps), assuming sphericity and similar composition for each selected diameter, and by fitting a Mie curve to the measured extinction cross sections at each specific wavelength.^28,51−55^ In brief, dried particles from the OFR were sampled after a VOC denuder. Particles were size-selected with an AAC, thus yielding a monodispersed particle size distribution. The monodispersed particles were directed into a photoacoustic (404 nm)–cavity ring-down spectrometer (404 nm)–broadband cavity-enhanced spectrometer (PAS-CRDS-BBCES) system and counted by a condensation particle counter (CPC, model 3752, TSI). The retrieval algorithm was limited to searching for *n* ≥ 1 and *k* ≥ 0.

## 3 Results and Discussion

### 3.1 Bulk Characterization of the BSOA_NO_3__ Using HR-Tof-AMS

**Figure 1:**
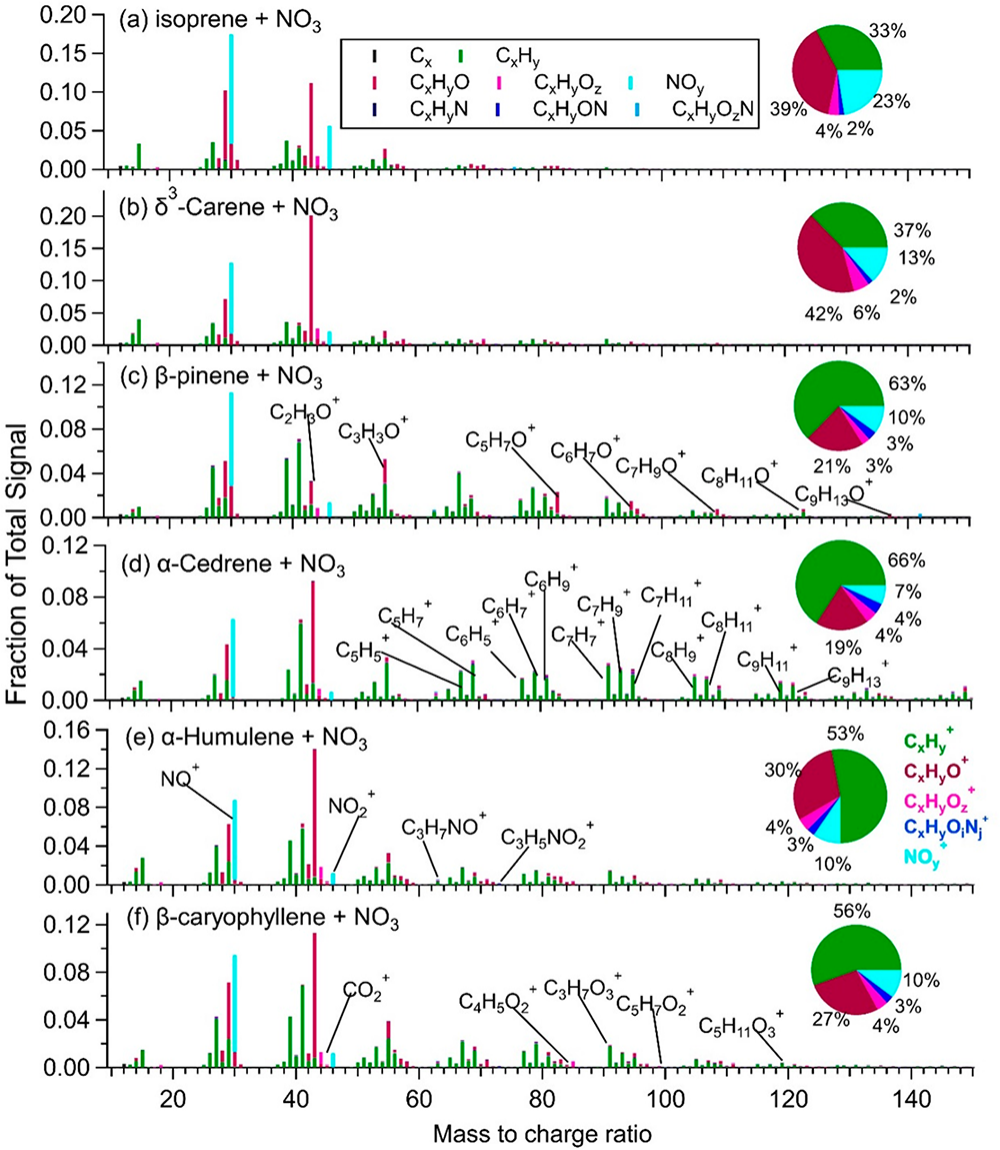
Chemical composition of the BSOA_NO_3__ measured by HR-Tof-AMS. The pie charts show the bulk chemical information, including organic-related fragments (grouped as C_*x*_H_*y*_^+^, C_*x*_H_*y*_O^+^, C_*x*_H_*y*_O_*z*_^+^, C_*x*_H_*y*_O_*i*_N_*j*_^+^, and NO_*y*_^+^, where *x*, *y*, *z*, and *j* ≥ 1, *i* ≥ 0). Large portions of hydrocarbon-like (indicated by C_*x*_H_*y*_^+^), less oxygenated fragments (indicated by C_*x*_H_*y*_O^+^), and nitrogen-containing fractions (indicated by C_*x*_H_*y*_O_*i*_N_*j*_^+^ and NO_*y*_^+^) are observed. The boxed legend is for the mass spectra, whereas the bold legend belongs to the pie chart. Ions of C_*x*_H_*y*_N, C_*x*_H_*y*_ON, and C_*x*_H_*y*_O_*z*_N in the mass spectra are categorized to C_*x*_H_*y*_O_*i*_N_*j*_^+^ in the pie chart.

The oxidation reactions of isoprene and terpenes with NO_3_· occur almost exclusively by the addition of the NO_3_· to the C=C double bond to form the most substituted nitrooxyalkyl radical.^56,57^ This nitrooxyalkyl radical reacts with O_2_ to create β-nitrooxyperoxy radical (RO_2_·) that further reacts with NO_3_·, hydroperoxyl radical (HO_2_·), and another RO_2_· to produce hydroxyl nitrate, carbonyl nitrate, and nitrooxyperoxide.^41^ Large RO_2_· species can undergo autoxidation to produce highly oxidized molecules^58−61^ or produce dimers through bimolecular reactions with another RO_2_·.^62,63^ The HR-Tof-AMS data of the SOA generated in this study show a high intensity (4.1–24.7%) at *m*/*z* 43 (C_2_H_3_O^+^, characteristic fragment of carbonyl compounds) and contain a considerable fraction (1.8–3.9%) of nitrogen-containing fragments (C_*x*_H_*y*_O_*i*_N^+^) (Figure 1 and Table S1), indicating that the production of carbonyls and ONs is favored during the NO_3_· oxidation of BVOCs, which is consistent with the known oxidation mechanism.^41^ Weak mass peaks at *m*/*z* 44 (CO_2_^+^) from carboxyl/acyl peroxide groups^64^ were detected (0.6–4.0%, Figure 1 and Table S1). These mass spectra of the SOA from the NO_3_· oxidation of BVOCs consist of a prominent C_*x*_H_*y*_^+^ ion signal (an indication of the hydrocarbon-like organic aerosol (HOA)) and C_*x*_H_*y*_O^+^ ion signals (a sign of carbonyl compounds), whereas the signature of higher-generation oxidation products (indicated by C_*x*_H_*y*_O_*z*_^+^ ions) is observed at trace levels. These features are common in the ambient semivolatile oxygenated organic aerosols (SV-OOAs) or LO-OOAs.^23,64−67^

Nitrogen-containing ions (C_*x*_H_*y*_O_*i*_N_*j*_^+^, NO^+^, and NO_2_^+^) comprise ∼17% (11–25%) of the combined organic and nitrate signals and are detected mainly as NO^+^ and NO_2_^+^ ions (7–23%) with a small amount of C_*x*_H_*y*_O_*i*_N_*j*_^+^ ions (2–4%) for all of the generated BSOA_NO_3__. In this study, the reactions were performed under dry conditions (RH < 5%), and no ammonium was detected in the SOA; therefore, nitrogen-containing fragments are predominantly from ONs. The characteristic fragment intensity ratio of NO^+^/NO_2_^+^ has been frequently used as an indicator of particulate organic nitrate, as this ratio is much higher for organic nitrates (usually assumed to be 10) than the ratio measured for inorganic nitrates, as determined by measuring it for NH_4_NO_3_.^19,68−70^ The NO^+^/NO_2_^+^ ratios in the mass spectra of the SOA ranged from 4.0 to 9.6 in this study, comparable to those observed for the SOA from the NO_3_· oxidation of isoprene, β-pinene, δ^3^-carene, and limonene.^15,70−74^ These ratios are higher than those of inorganic nitrates (2.2 for NH_4_NO_3_), further supporting the formation of ON products. The N/C ratio of the SOA formed from the β-pinene + NO_3_ reaction averaged 0.077, which is in good agreement with the reported values of 0.070 to 0.076 in previous studies.^15,74,75^

### 3.2 Complex Refractive Index of the Generated BSOA_NO_3__

**Figure 2:**
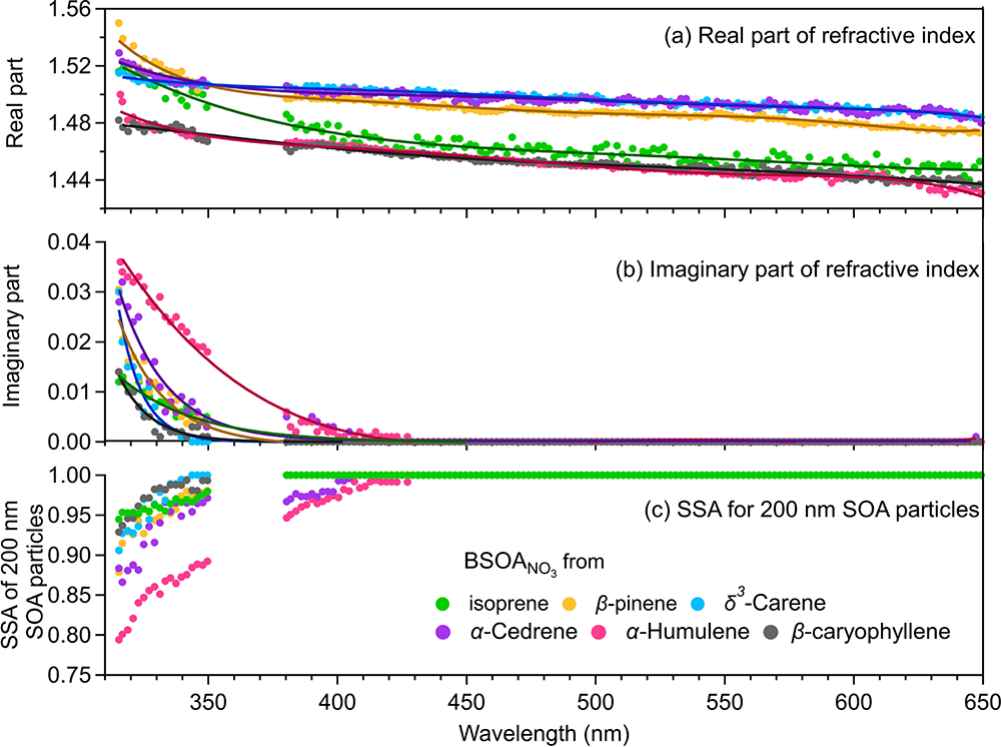
Wavelength-dependent optical properties of the BSOA_NO_3__. The real part (a) and imaginary part (b) decrease with increasing wavelength. The *k* is observed only at the deep UV wavelength range. (c) Single scattering albedo (SSA) values were calculated for 200 nm spherical particles. Data for β-pinene, β-caryophyllene, and the α-humulene-derived SOA are taken from experiments with a N_2_O_5_/VOC ratio of 1.2, 10.7, and 8.8, respectively. The results of isoprene, δ^3^-carene, and α-cedrene are obtained under a N_2_O_5_/VOC ratio of 1.3, 1.9, and 5.8, respectively.

Only a few previous studies have investigated the RI of the SOA produced from NO_3_· oxidation.^35−37^Figure 2 shows the RI of the BSOA_NO_3__ across most of the solar wavelength range (315–650 nm). A comparison of our results and the literature data is shown in Figure S5. To the best of our knowledge, this is the first set of wavelength-resolved RI results for the BSOA_NO_3__ over such a wide wavelength range based on online measurements. The real part of the RI (*n*) of the BSOA_NO_3__ in this study varied between 1.43 and 1.55. The real RI for the generated SOA exhibits a slight spectral dependence with *n* values that decrease with increasing wavelength. This weak wavelength dependence is similar to that observed for the SOA from the OH· oxidation of β-pinene^33^ and the ozonolysis of monoterpenes.^31^ Moreover, the results from the BBCES are consistent with those from an independent CRD measurement. The value of *n* at 404 nm for the SOA of isoprene + NO_3_ (1.472 ± 0.007) from our study is similar to those (1.455_–0.023_^+0.023^ to 1.468_–0.027_^+0.025^) measured for the SOA from isoprene + O_3_ + NO_*x*_ in the presence/absence of sulfur dioxides.^34^ However, the values of *n* at 532 nm for the SOA from β-pinene + NO_3_ (1.486 ± 0.001) and δ^3^-carene + NO_3_ (1.493 ± 0.001) are much lower than that (1.578) of limonene + NO_3_,^35^ indicating that the real RI of the BSOA_NO_3__ highly depends on the VOC precursor. Varma et al.^37^ studied the NO_3_-initiated oxidation of β-pinene under dry conditions using the BBCES at the SAPHIR atmospheric simulation chamber. They determined an *n* value of 1.61 ± 0.03 between 655 and 687 nm, assuming no absorption. In our study, the wavelength range was limited to 650.7 nm. The *n* value for the BSOA_NO_3__ from β-pinene at this wavelength is 1.474 (±0.001), which is substantially lower than those from the SAPHIR experiments. The SAPHIR experiments were conducted at much lower VOC levels (<18 ppbv) and for a longer period (∼1 h) compared with this study (>40 ppbv, 162 s). These differences in the experimental conditions may lead to a differences in the SOA formation that will further affect the real RI.

Previous studies determined a near-zero imaginary part of the RI (imaginary RI, *k*) of the SOA produced by the photooxidation/ozonolysis/OH oxidation of BVOCs under NO_*x*_-free conditions for the atmospherically relevant wavelength region (λ > 300 nm), especially in the visible range.^3,27,32,33,51^ In this study, we determined the optical properties of the BSOA_NO_3__ in the short UV wavelength range (315–350 nm) using our unique UV channel (BBCES_UV_). Most of the generated SOA absorbs slightly in the deep UVA wavelength range, and *k* decreases with increasing wavelength (Figure 2 and Table S5), which is the typical behavior of BrC. The *k* values obtained from all of the oxidation experiments are 0.003 to 0.046 at 316 nm and 0.001 to 0.039 at 330 nm. Above 390 nm, very weak light absorption was detected, in line with previous findings for the BSOA_NO_3__.^34,35,37^ The retrieved *k* in the UV range is similar to that observed for the SOA produced by the photooxidation of aromatic compounds (e.g., toluene and *m*-xylene) and higher than that of the SOA generated by the ozonolysis of α/β-pinene.^31,76^ The *k* of the BSOA_NO_3__ obtained in this study is much lower than that for the ammonia-aged biogenic SOA^51^ and biomass burning aerosols (Figure S5).^38,77−80^ Recently, the optical properties of the SOA from the NO_3_· oxidation of unsaturated heterocyclic VOCs were studied.^81^ The values of *k* for pyrrole and the thiophene-derived SOA range between 0.002 and 0.017 at 375 nm. These values are higher than those measured for the BSOA_NO_3__ in this study. Previous studies for isoprene + NO_3_ have reported an *k* value of 0.0001 at 375 nm when sulfur dioxide is added during the oxidation process.^34^ In this study, the *k* value of the SOA from isoprene + NO_3_ is 0.005 (±0.006) at 349.4 nm, and it is essentially zero in the longer wavelength range (380–650 nm). The imaginary RI of the BSOA_NO_3__ from α-humulene is the largest among all of the studied systems. As discussed in Sections 3.3–3.5, the absorption is controlled by the ONs in the SOA, and ONs that have a carbonyl-adjacent nitrate group exhibit stronger absorption compared with other types of ONs. The high N_2_O_5_/VOC ratio in the α-humulene + NO_3_· experiment (8.8) favors the formation of ONs. Moreover, α-humulene has three substituted C=C bonds (two, one, one, one, and two for isoprene, β-pinene, δ^3^-carene, α-cedrene, β-caryophyllene, respectively) that can form stable (and do not favor cyclize reactions) ONs with a carbonyl adjacent to the nitrate group. This results in a stronger absorption in the BSOA_NO_3__ from α-humulene as compared with the other types of BSOA_NO_3__.

The single scattering albedo (SSA) (SSA = scattering/extinction) is frequently used in climate models. The SSA data for the BSOA_NO_3__ in this study were calculated for 200 nm particles based on the Mie theory using the retrieved refractive index. The SSA is 1 for all BSOA_NO_3__ above 425 nm. In the UV ranges, the SSA increases from 0.80 to 1 with increasing wavelength. These values are higher than those obtained for the SOA produced from aromatics^32,76,82^ and the aerosol derived from biomass burning.^80,83^ Overall, the BSOA_NO_3__ is not a significant BrC contributor. However, it does absorb substantially between 280 and 300 nm, meaning it is photochemically active in the UVA range, which likely induces condensed-phase photochemistry.

### 3.3 Linking Light Absorption with Chromophores

**Figure 3:**
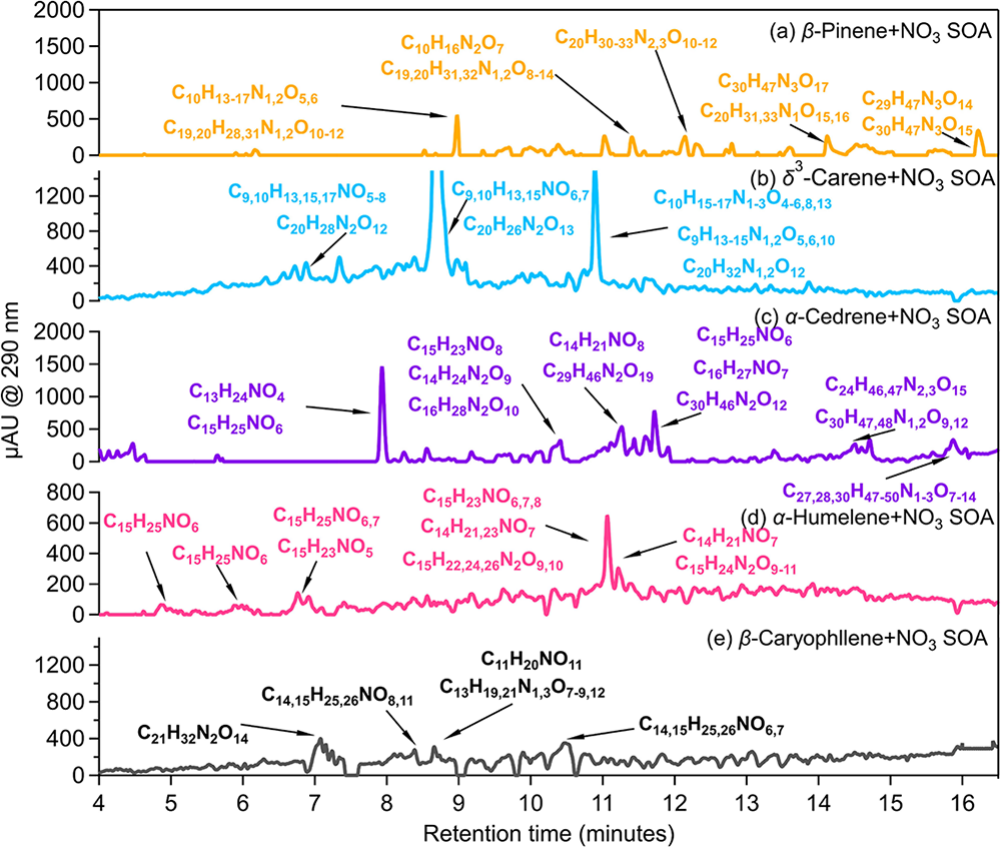
Possible formulas of absorbing compounds detected in the BSOA_NO_3__ by HPLC-PDA-HESI/HRMS. ONs are responsible for the absorption peaks observed by the PDA. Data for the β-pinene-, β-caryophyllene-, and α-humulene-derived SOAs are taken from experiments with a N_2_O_5_/VOC ratio of 1.2, 10.7, and 8.8, respectively.

The nighttime reactions of BVOCs with NO_3_· lead to the formation of secondary BrC, which absorbs at short wavelengths. Therefore, it is essential to identify the compounds that are responsible for the observed absorption. Figure 3 shows the UV–vis chromatograms at 290 nm (blank corrected) as detected by the ultraperformance liquid chromatography–photodiode array (UPLC-PDA) performed in parallel with heated electrospray ionization/high-resolution mass spectrometry (HESI/HRMS). We present absorption at 290 nm to provide a better signal-to-noise ratio. Significant absorption was observed at a retention time (RT) of 9.0 min in the BSOA_NO_3__ from β-pinene. High abundances of monomers and dimers with formulas of C_10_H_13–17_O_5,6_ and C_19,20_H_28,31_N_1,2_O_10–12_ were found in the HESI/HRMS chromatograms. The UV–vis absorption peak at ∼290 nm, which is the absorption feature of carbonyls or ONs,^84,85^ is coincident with the result from HR-Tof-AMS in that large amounts of carbonyls or ONs were produced in the BSOA_NO_3__. We extracted the wavelength-dependent absorption spectra for the chromatograms shown in Figure 3 and compared them to those of typical nitrate-containing organics and carbonyls (Figure S3). The spectra obtained from the BSOA_NO_3__ showed an absorption maximum at ∼290 nm within the wavelength range of 240–340 nm, which is similar to that of carbonyls and organic nitrates. This further supports the formula’s assignment from the mass spectrometer and illustrates that carbonyl ONs are responsible for light absorption, although it is not possible to differentiate the contribution of each compound due to their overlapping elution times in the UPLC. In the other types of BSOA_NO_3__ studied, the light absorption was attributed to the most abundant ON dimers and oligomers. Interestingly, two C_15_H_25_NO_6_ isomers in the BSOA_NO_3__ from α-humulene are potential light -absorbers, whereas only one of the four isomers of C_15_H_24_N_2_O_9_, the dominant species in the mass spectra, showed detectable light absorption (Figure S6). This indicates that different isomers can have quite different lifetimes regarding atmospheric photolysis. Only a few light-absorbing species with weak absorption were identified in the BSOA_NO_3__ from β-caryophyllene (Figure 3e), consistent with the result from the online BBCES measurements.

### 3.4 Influence of the N_2_O_5_/VOC Ratio on the Chemical Composition and RI

**Figure 4:**
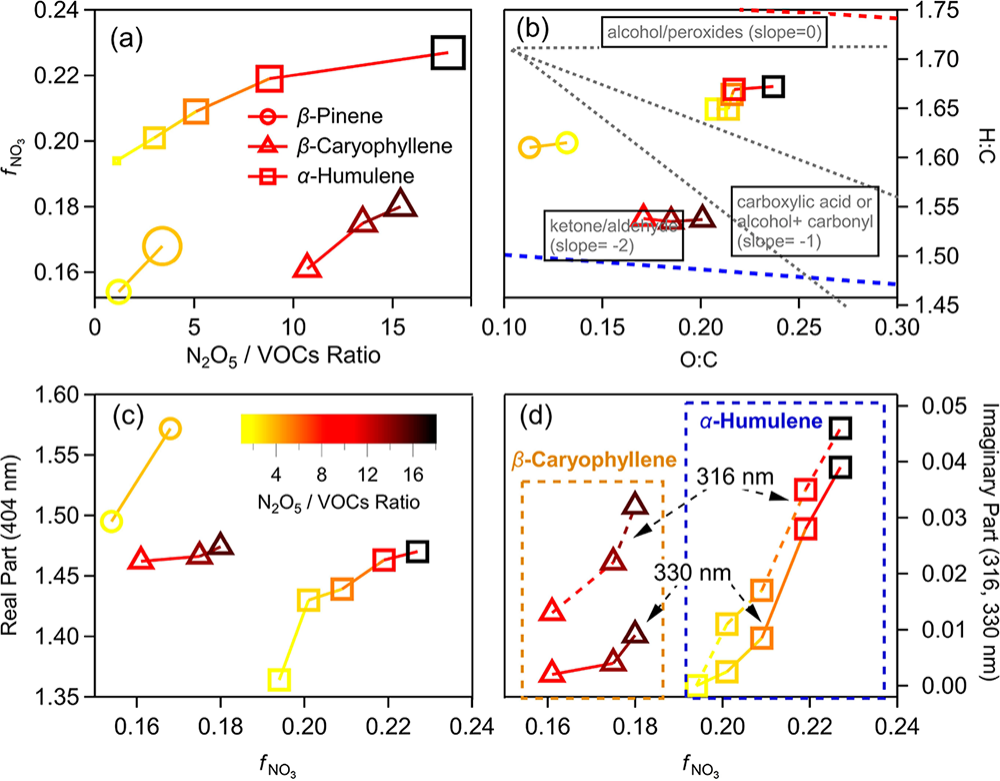
Influence of the initial N_2_O_5_/VOC ratio on the chemical–physical properties of the BSOA_NO_3__ from β-pinene (○), β-caryophyllene (△), and α-humulene (□). The symbol’s color indicates the initial N_2_O_5_/VOC ratio, and the symbol’s size in panel a represents the effective particle density. With the increasing initial N_2_O_5_/VOC ratio, the nitrate fraction (*f*_NO_3__) and the particle’s effective density (a), the elemental ratios (b), the real refractive index at 404 nm (c), and the imaginary refractive index at 316 and 330 nm (d) increased.

Faxon et al.^14^ measured the chemical composition of the SOA from the NO_3_· oxidation of limonene using a high-resolution time-of-flight chemical ionization mass spectrometer combined with a filter inlet for gases and aerosols to measure. They found that the chemical composition of the SOA (e.g., thermally unstable dimers) changed dramatically with the initial N_2_O_5_/limonene ratio. In this study, we produced the SOA from β-pinene, β-caryophyllene, and α-humulene at different N_2_O_5_/VOC ratios (Table S1). Under higher initial N_2_O_5_/VOC ratios, more NO_3_· was involved in the reaction with BVOCs and intermediates or taken up by particles to react with organic species (Table S4). Thus the SOA contains more nitrate groups, as was observed by HR-Tof-AMS (Figure 4a). This was also confirmed by the AMS fragment analysis, where the contribution of NO_*y*_^+^, which originated from ONs in the SOA, increased with the initial N_2_O_5_/VOC (Figure S7). The studied terpenes have C=C double bonds with more than one ring. Thus the first-generation oxidation products from NO_3_·-initiated oxidation may still contain a C=C double bond that can further react with NO_3_· to generate products with multiple nitrate groups. The RI of the SOA also changed under different initial N_2_O_5_/VOC ratios, with a higher real part and imaginary part of the RI under higher N_2_O_5_/VOC ratios (Figure S8).

Both the H/C and O/C ratios, the particle effective density, and the nitrate fraction (*f*_NO_3__) in the SOA observed by HR-Tof-AMS increased with increasing N_2_O_5_/VOC ratios, as shown in Figure 4b–d, confirming the functionalization (e.g., −OH or −OOH addition to the C=C bonds) during the oxidation by NO_3_·. The Lorentz–Lorenz relationship () correlates the real RI (*n*) to the mean polarizability (α), the particle effective density (ρ), and the average molecular weight (MW) of the SOA. The mean polarizability can be estimated by the additive group contribution method.^86,87^ The enhanced functionalization under higher N_2_O_5_/VOC ratios significantly increases the H/C and O/C ratios and the nitrate fraction and therefore increases the mean polarizability of the SOA, in combination with the increased effective density, causing an increase in the real RI of the BSOA_NO_3__ under higher N_2_O_5_/VOC ratios. HPLC-PDA-HESI/HRMS results have revealed that ONs are responsible for the observed light absorption. Under higher initial N_2_O_5_/BVOC ratio conditions, the *f*_NO_3__ of the produced SOA is higher, indicating more abundant ONs in the SOA. As a result, a larger imaginary RI is expected. In urban conditions, the NO_3_· production rate is enhanced, whereas BVOC emissions can be relatively lower. Thus the produced BSOA may show more light-absorbing ability over and downwind of cities.^88^

### 3.5 Optical Properties Evolution upon Photochemical Aging

Chemical characterization shows that ONs comprise a significant fraction of the light-absorbing BSOA_NO3._ Therefore, it is important to understand how the optical properties change during daytime OH-dominated oxidation. To address this important question, we exposed the monoterpene (β-pinene) and sesquiterpene (α-humulene) BSOA_NO_3__ to an equivalent of 1 day of aging by OH· in a PAM OFR. As shown in Table S6, a slight decrease in the *f*_NO_3__ was observed (0.201 to 0.196 and 0.206 to 0.192 for β-pinene and α-humulene, respectively), indicating that the particulate ONs are resistant to OH· aging, which is consistent with previous findings for the BSOA_NO_3__ from β-pinene.^15^ For the BSOA_NO_3__ from β-pinene, whereas the O/C ratio of the OH·-aged SOA increased slightly, the H/C ratio decreased, indicating the H-abstraction reaction during OH· aging. Upon OH· aging of the BSOA_NO_3__ from α-humulene, a slight increase in the H/C (∼0.005) and O/C ratios (by 0.022) was observed, indicating functionalization (OH· addition), possibly due to the remaining unsaturated C=C bonds in the SOA, which favors the addition of functional groups.

**Figure 5:**
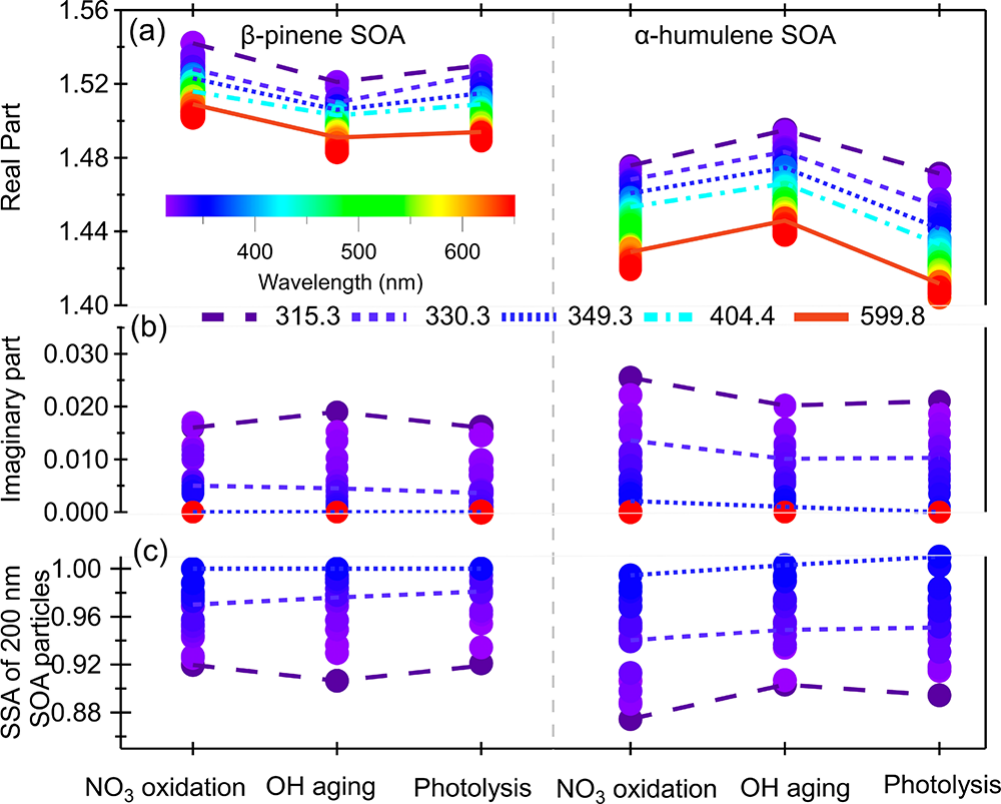
Modification of the RI and single scattering albedo (SSA) of the BSOA_NO_3__ by OH· aging and photolysis. (A,B) Evolution of the broadband RIs of the BSOA from the NO_3_· oxidation of β-pinene and α-humulene after 24 h of equivalent ambient OH· exposure time or 1.7 × 10^14^ photons cm^–2^ photolysis at 254 nm. (C) SSA transformations for 200 nm SOA particles at UV wavelengths (315–349 nm). Changes in the RI and SSA at 315.3 (purple), 330.3 (light blue), 349.3 (blue), 404.4 (cyan), and 599.8 (red) are displayed as lines.

In Figure 5, we show the RI evolution from photochemical aging. The real part at 315.3, 330.3, 349.3, 404.4, and 599.8 nm and the imaginary part at 315.3, 330.3, and 349.3 nm are highlighted. For the BSOA_NO_3__ from β-pinene, the real RI decreased slightly when the SOA was processed with photolysis and photochemical aging, whereas no significant change in the imaginary RI was observed. For the BSOA_NO_3__ from α-humulene, the real RI of the SOA increased during OH· aging, and it is resilient to photolysis. The imaginary RI decreased during both the photolysis and the OH· aging process. Therefore, the SSA for 200 nm particles increased. For the BSOA_NO_3__ from β-pinene, the real RI decreased slightly, possibly due to the loss of ON moieties that efficiently scatter light. The absorption of the SOA is linked to specific ONs (e.g., C _10_H_13–17_NO_5,6_ and C_19,20_H_28,31_N_1,2_O_10–12_), as shown in Figure 3. Because of the deactivation of the C–H bonds by adjacent functional groups, these highly functionalized ONs have fewer H–C bonds available for H abstraction by OH· oxidation, making them more resistant than less functionalized compounds. Thus the imaginary RI does not significantly change during the OH· aging experiments. Moreover, the interaction between the carbonyl and the nitrate functional groups will induce strong light absorption. A previous study by Draper et al. has found that ONs with a carbonyl adjacent to the nitrate group can be produced through left scission reactions of nitroxyalkoxyl radicals that are produced by the NO_3_· radical oxidation of unsaturated VOCs.^13^ The β-pinene molecule has only one substituted C=C double bond to form products with a carbonyl adjacent to the nitrate group. Moreover, these products rapidly cyclize and further react in particles to form acetal heterodimers and heterotrimers, leading to a loss of the carboxyl adjacent to the nitrate groups (Figure S9).^57^ Thus no significant strong absorbing species will be produced in the BSOA_NO_3__ from β-pinene. Moreover, in the ON produced from the NO_3_· oxidation of β-pinene, the carbonyl group is further away from the nitrate functional group, or there is a hydroxyl functional group adjacent the nitrate functional group.^57,74^ The lack of interaction between carbonyl and nitrate functional groups seems to induce negligible light absorption. This weak light-absorbing ability of the BSOA_NO_3__ also suggests that its photolysis will be insignificant, which is in line with the previous finding that the ON fraction of the BSOA_NO_3__ from β-pinene was resistant to photochemical aging.^15^ As a result of the significant decrease in the real part and the small change in the imaginary part upon OH· aging, the SSA decreased in the UVA range, indicating that the aged SOA can have a relatively stronger warming effect. The α-humulene molecule has three substituted C=C double bonds that form products with a carbonyl adjacent to the nitrate group. The carbonyls adjacent to the nitrate groups are further away from the hydroxyl group, which does not favor the cyclize process, stabilizing in the particle phase. The coupling of these two functional groups in the BSOA_NO_3__ from α-humulene can enhance the light absorption, as confirmed by the relatively high imaginary RI, resulting in the higher photolysis efficiency of ONs. The photolysis proceeds by releasing NO_2_ and forming compounds with fewer or even no nitrate groups. Photolysis at UVA will also decompose carbonyls, which would have an additional photobleaching effect. Thus upon photolysis, both the real part and the imaginary part of the RI decreased, resulting in an overall increase in the SSA. On the basis of the change in absorption, we calculated the photolysis lifetime of absorbing ON in the BSOA_NO_3__ to be 6.2 h (Text S3), assuming no phase separation, which may affect the aging. Assuming no synergetic effect between photolysis and OH· aging and excluding the photolysis-induced decrease in absorption, the OH· aging also bleached the particles with a lifetime of 38.8 days. For the α-humulene-derived BSOA_NO_3__, bleaching by photochemical aging (including OH· aging and photolysis) is governed by photolysis, and the lifetime is ∼6 h. These results from the β-pinene and α-humulene aging experiments indicate that the effect of photochemical aging on the optical properties (refractive index and SSA) of the BSOA_NO_3__ largely depends on the specific chemical nature of the ONs and their precursors, leading to a more complicated picture than just “bleaching” or “browning”.

## 4 Atmospheric Implications

The study provides the chemical composition and optical properties of the SOA produced during the NO_3_· oxidation of terpenoids. The BBCES-CRD measurements show that the nighttime reactions studied here form BrC, which weakly absorbs light in the UVA range. According to the framework recently introduced by Saleh et al.,^89^ the produced BSOA_NO_3__ falls into the category of very weakly absorptive BrC. The UPLC-PDA-HRMS analysis confirmed that ONs are responsible for light absorption. In urban and suburban areas that are affected by anthropogenic pollution and high BVOC emissions, the high NO_*x*_ and O_3_ promote NO_3_· production. This could also result in a high NO_3_·/VOC ratio, which favors BrC that contains a high fraction of ONs. Although the light absorption of the BSOA_NO_3__ is weak, the SOA formation from the nighttime NO_3_· oxidation of BVOCs is efficient, especially in regions where massive anthropogenic emissions mix with BVOCs. Therefore, the BSOA_NO_3__ can have a measurable impact on the aerosol UVA absorption, which could further affect the climate and air quality on a regional scale.

The BSOA_NO_3__ from α-humulene has an equivalent photolysis lifetime longer than 6.2 h (Text S3). Because of their weak light-absorbing properties, the photolysis of the ONs in the β-pinene BSOA_NO_3__ is negligible (Figure 5). Previous studies have tested the bulk hydrolysis properties of the BSOA_NO_3__ from α- and β-pinene.^16,74^ The hydrolysis lifetime varies between 0.02 and 8.8 h, depending on the precursor VOCs, oxidant type, aerosol acidity, relative humidity, and more. Although the lifetime is short, the ON from NO_3_· oxidation has a low hygroscopicity, and only a small fraction (≤17%) can undergo hydrolysis.^16^ Taking all of these factors into consideration, we suggest that the BSOA_NO_3__ generated from β-pinene and α-humulene at night would survive into the morning hours or longer and would scatter and absorb the incoming solar radiation and sequester NOx. These results indicate that ONs produced from the NO_3_· oxidation of β-pinene and α-humulene may serve as NOx reservoirs or permanent NO_*x*_ sinks in the atmosphere, which is consistent with previous findings.^15^ We note that previous studies by Nah et al. have found that the particle-phase ONs in the BSOA_NO_3__ from α-pinene evaporate during photochemical aging.^15^ The significant photolysis of the α-pinene BSOA_NO_3__ could dramatically change its optical properties, as it behaves differently compared with the BSOA_NO_3__ from α-humulene and β-pinene. These results indicate that ONs in the BSOA_NO_3__ produced from terpenes can serve as either temporary or permanent NOx sinks depending on the precursor. This finding has significant implications for NO_*x*_ and O_3_ budgets in areas with high emissions of monoterpenes and sesquiterpenes, such as the Southeastern United States, Northern Europe, and Southeast Asia. We suggest incorporating these processes into the current modeling strategies to improve NO_*x*_ and O_3_ simulations.

This study focused on the ON production by the NO_3_· oxidation of BVOCs, the optical properties of the resulting BSOA, and the evolution of their chemical and physical properties during the transition from night to day. The link between the BSOA_NO_3__ formation mechanism, its chemical and physical properties, and the dynamic evolution was illustrated for both laboratory simulations and ambient aerosols. Photolysis and OH· aging were studied here under low relative humidity (37.5%) conditions, and the possible role of the hydrolysis of the ONs was not investigated. It is also noted that isomers with the same formula have different light-absorbing properties and lifetimes. Therefore, isomer-specific studies may be helpful for understanding the bulk chemical and physical properties (e.g., hydrolysis and oxidation) of ONs and the SOA. Obviously, RO_2_· chemistry plays a role in determining the changes to the chemical and optical properties of the SOA. In this study, we focused on one set of (extreme) conditions. More detailed studies and additional modeling efforts will be conducted to understand how different RO_2_· regimes affect these changes and how these translate to different atmospheric chemical regimes.

## Supporting Information Available

Additional description of methods; additional tables showing details of the SOA generation, chemical and optical results of the BSOA_NO_3__, and the model simulation results in the OFR; additional figures showing the experimental setup, absorption spectra of ONs and carbonyls from the literature and those of compounds of interest in the BSOA_NO_3__, RI results for the BSOA_NO_3__ and from the literature, and the influence of the N_2_O_5_/VOC ratio on the chemical composition and RI (PDF)
