# Supplementary material for: Optical Properties of Secondary Organic Aerosol Produced by Nitrate Radical Oxidation of Biogenic Volatile Organic Compounds
Source: Environ Sci Technol. 2021 Feb 17;55(5):2878–89. doi: 10.1021/acs.est.0c06838 (PMC8023652; doi:10.1021/acs.est.0c06838)
Supplement: Supplementary file 1 — es0c06838_si_001.pdf [file es0c06838_si_001.pdf]

Supporting information for

Optical Properties of Secondary Organic Aerosol Produced by Nitrate Radical Oxidation of Biogenic Volatile Organic Compounds

Quanfu He,[†] Sophie Tomaz,[‡] Chunlin Li,[†] Ming Zhu,[⊥,§] Daphne Meidan,[†] Matthieu Riva,[‡] Alexander Laskin,[○] Steven S. Brown,[#,∥] Christian George,[‡] Xinming Wang,[⊥,§,∇] and Yinon Rudich[†,*]

[†] Department of Earth and Planetary Sciences, Weizmann Institute of Science, Rehovot 76100, Israel

[‡] Univ Lyon, Université Claude Bernard Lyon 1, CNRS, IRCELYON, F-69626, Villeurbanne, France

[⊥] State Key Laboratory of Organic Geochemistry and Guangdong Key Laboratory of Environmental Protection and Resources Utilization, Guangzhou Institute of Geochemistry, Chinese Academy of Sciences, Guangzhou 510640, China

[§] University of Chinese Academy of Sciences, Beijing 100049, China

[○] Department of Chemistry, Purdue University, West Lafayette, Indiana 47907, United States

[#] Chemical Sciences Division, Earth System Research Laboratory, National Oceanic and Atmospheric Administration, 325, Broadway, Boulder, CO 80305, USA

[∥] Department of Chemistry, University of Colorado, 216 UCB, Boulder, CO 80309, USA

[∇] Center for Excellence in Urban Atmospheric Environment, Institute of Urban Environment, 11 Chinese Academy of Sciences, Xiamen 361021, China

Correspondence to: Yinon Rudich (yinon.rudich@weizmann.ac.il)

This supporting information file has 24 pages, including

6 texts: Text Sa-S6

6 tables: Table S1-S6

9 figures: Figure S1-S9

35 references

Supporting Methods (Text S1-6)

Text S1. Synthesis N_2O_5 crystals

Text S2. N_2O_5 measurement using cavity ring-down spectroscopy (CRDS)

Text S3. Box modeling of the reactions in the OFR_{NO_3}.

Text S4. Estimation of the effective photolysis time in the PAM reactor.

Text S5. Offline chemical composition analysis of $BSOA_{NO_3}$.

Text S6. Refractive index (RI) of BSOA retrieved from BBCES-PAS-CRDS measurements

Supporting Figures (Figures S1-S6)

Figure S1. Experimental setup.

Figure S2. Determination of the equivalent photolysis time in the PAM.

Figure S3. Absorption spectra of various reference compounds and compounds of interest in the $BSOA_{NO_3}$.

Figure S4. Box model simulated fate of N_2O_5, NO_3•, and *β*-pinene/*α*-humulene.

Figure S5. The refractive index for $BSOA_{NO_3}$ generated in this study and literature results.

Figure S6. The absorption and extracted ion chromatograms for $BSOA_{NO_3}$ generated from the *α*-humulene.

Figure S7. Influence of N_2O_5/VOC ratio on the fragments composition of $BSOA_{NO_3}$.

Figure S8. Influence of the N_2O_5/VOC ratio on the complex refractive index of the $BSOA_{NO_3}$.

Figure S9. The NO_3• initialed oxidation mechanism of *β*-pinene and *α*-humulene.

Supporting Tables (Tables S1-S6)

Table S1. SOA generation and chemical-physical characteristics of the produced $BSOA_{NO_3}$.

Table S2. Estimated reaction kinetics for NO_3• and N_2O_5 in the OFR_{NO_3}

Table S3**.** List of reactions and their rate constants for the BVOCs+NO_3 simulation.

Table S4. Loss of NO_3• and N_2O_5 in the OFR_{NO_3}.

Table S5. Refractive index measured for different types of $BSOA_{NO_3}$ in this study.

Table S6. The effect of OH radical aging and photolysis on the chemical composition of $BSOA_{NO_3}$.

Supporting References (41)

Supporting Methods

Text S1. Synthesis N_2O_5 crystals

N_2O_5 crystals were used as NO_3• sources. In detail, a flow of NO (≥99.9 %, Verdichtetes Gas) is mixed with ultrahigh purity O_2 (≥99.999%, Air Liquid UK Limited.) in a glass bulb (3 L) to produce NO_2 (S1) by the Bodenstein reaction. The NO_2 then reacts with O_3 generated online by an ozone generator (Pacific Ozone Tech, USA) in a Teflon tube (1" OD, 75 cm length) to produce NO_3• (S2) which further react with NO_2 to produce N_2O_5 (S3). After the Teflon reactor, the output which exclusively containing N_2O_5 is fed to a glass trap immersed in a mixture of dry ice and ethanol to collect N_2O_5 crystals.

$2NO + O_2 \leftrightarrow 2NO_2$ S1

$NO_2 + O_3 \rightarrow NO_3\bullet + O_2$ S2

$NO_2 + NO_3\bullet \leftrightarrow N_2O_5$ S3

Gaseous N_2O_5 was carried out by a gentle ultrapure N_2 flow (10−140 mL min^{-1}) from the cold trap followed by mixing with other gases in the oxidation flow tube reactor (OFR).

Text S2. N_2O_5 measurement using cavity ring-down spectroscopy (CRDS)

The initial mixing ratio of N_2O_5 brought out by the nitrogen flow was measured using a heated (85 °C) cavity ring-down spectrometer (CRDS) working at 662 nm. The NO_3• produced by thermal decomposition of N_2O_5 has a strong absorption cross section ($\sigma_{abs\text{-}NO_3}$ = 1.7 × 10^{-17} cm^2 $molec^{-1}$) at this wavelength. Gaseous absorption measurement by CRDS has been extensively described before.[25-27] The CRDS consists of a temperature-controlled single-wavelength laser diode, a temperature-controlled Teflon optical cavity equipped with two high-reflectivity mirrors at both ends which form a stable optical resonator, and a photomultiplier as a detector. Gas absorption coefficient (α_{abs}) can be determined by measuring the light decay time for an empty cavity (τ_0) and a filled cavity (τ), as shown in Equation S4:

$$\alpha_{abs} = \frac{L}{lc}\left(\frac{1}{\tau} - \frac{1}{\tau_0}\right) \quad \text{S4}$$

where L is the optical length (0.979 m), l is effective cavity length (0.752 m), and c is the light velocity (2.99792458 × 10^8 m s^{-1}). With known absorption cross section of NO_3• (σ_{abs}) at 662 nm, the concentration (C) of the gas can be calculated as S6:

$$C = \frac{\alpha_{abs}}{\boldsymbol{\sigma_{abs}}} \quad \text{S5}$$

N_2O_5 is measured indirectly via NO_3• following thermal dissociation of N_2O_5 at 85 °C. NO_3• produced from N_2O_5 was measured by titrating away NO_3• with NO:

$NO_3\bullet + NO \rightarrow 2NO_2$ S6

Titration of NO_3• was achieved in a warm (85 °C) Teflon tube (Length: 3.0 m, ID: 6.0 mm) upstream of the CRDS. The residence time was 15 s, which was long enough to reach equilibrium. At fixed N_2O_5 flow (10-140 mL min^{-1}), NO was increased slowly until the residual NO_3• concentration was lower than 10 times of the CRDS detection limit (0.2 pptv). Based on the added NO and dilution ratio, the N_2O_5 concentration could be calculated.

Text S3. Estimation of the effective photolysis time in the PAM reactor.

The rate constant of a photochemical reaction, J_{PAM}, for a compound in the SOA in the PAM can be described as

$$J_{PAM} = \int \sigma(\lambda)\, \varphi(\lambda) F(\lambda) d\lambda \qquad \text{S7}$$

Where σ(λ), ψ(λ), F(λ) are the absorption cross section (cm^2 $molec^{-1}$), the quantum yield for the process of interest, and the actinic flux (photon cm^{-2} s^{-1} nm^{-1}) at one wavelength (λ), respectively. In the ambient, the effective photochemical rate constants ($J_{ambient}$) can be calculated for the daily averaged actinic flux

$$J_{ambient} = \int \sigma(\lambda)\, \varphi(\lambda) F(\lambda, mean) d\lambda \qquad \text{S8}$$

Thus, under a specific residence time (t_{PAM} =252 s) in the PAM, the equivalent photolysis time (T) in the PAM can be calculated as

$$T = \frac{J_{PAM}}{J_{ambient}} \times t_{PAM} \qquad \text{S9}$$

Although the gas absorption cross-section and quantum yields are known for a number of simple organic compounds, there is limited information for the multifunctional molecules in the SOA. Moreover, only a fraction of the SOA can undergo photolysis reactions, leading to a change in the SOA. In this work, we used a simplified approach to calculate the photolysis rate of the SOA based on the compounds which have the strongest absorption as detected by the HPLC-PDA-HESI/HRMS (Figure 2). For example, chromatograms of $BSOA_{NO_3}$ from α-humulene shown the strongest absorption at the retention time of 11.1 min. The wavelength-dependent absorption (linear correlated with absorption cross section) was then applied for the J estimation, which resembles an upper limit. The quantum yields for the photolysis reactions of SOA constitutes are unknown. In the SOA generated, a lot of carbonyl and nitrate groups were detected. Moreover, the extracted absorption spectra (Figure S8) suggest that a lot of carbonyl nitrates will be produced. Thus a unity quantum yield of 0.9 recommended by previous studies[1,2] for carbonyl nitrate was employed in this study. The integrated photon flux at 254 nm in the PAM reactor was estimated to be 1.7×10^{14} photon cm^{-2} s^{-1} based on the measured OH• concentration using a photochemical model.[3,4] Light spectra in the PAM were recorded by a spectrometer, which was used to calculate the photon flux at other wavelengths. The daily averaged actinic flux on December 17th, 2019, in Rehovot, Israel, was calculated (Figure S14) using the Tropospheric Ultraviolet and Visible (TUV) model from NCAR. During the calculation, the upper limit is arbitrarily set to 340 nm because of negligible absorption cross over this wavelength. Based on equation S13-16, the equivalent photolysis time was calculated to be 0.83 hrs in the PAM reactor. After such a photolysis period, integrated wavelength-dependent absorption of $BSOA_{NO_3}$ from a-humulene decreased by 13% as derived from the refractive index. Thus the equivalent photolysis lifetime of $BSOA_{NO_3}$ from α-humulene is 6.2 hrs. We should note that this value is the lower limit as we used carbonyl nitrate as a photolysis model compound for calculation. In the SOA generated, a lot of other compounds have smaller absorption cross-sections and quantum yields, especially in the atmospheric-relevant wavelength range. Thus a lower photolysis rate and a longer photolysis lifetime are expected.

Text S4. Box modeling of the reactions in the OFR_{NO_3}.

Text S4.1. Production and Fate of NO_3• and N_2O_5.

In the gas phase, NO_3• produced from N_2O_5 reacted with BVOCs. Products from this oxidation process and further reactions generated organic particles through the homogeneous nucleation process. Moreover, heterogeneous reactions occurred when the produced organic particles mixed with gaseous N_2O_5 and NO_3• in the OFR_{NO_3}. Previous studies have pointed out that N_2O_5 and NO_3• involve different heterogeneous reaction pathways. N_2O_5 mainly undergoes hydrolysis on particle surfaces to produce nitric acid, and it can be an efficient nitrating agent when it was up-taken by organic

surface.[5,6] NO_3 radicals can initiate a series of oxidation reactions in the presence of NO_2 and O_2.[7,8] The overall sink of NO_3• and N_2O_5 in the experiment can be simply described by the gas phase reaction and pseudo-first-order loss to the particle and reactor surface S10-12:[9,10]

$$\frac{d[\mathrm{G}]}{dt} = -(k_g[\mathrm{G}] + k_p[\mathrm{G}] + k_w[\mathrm{G}]) \quad \text{S10}$$

$$k_p = \frac{\gamma_{eff} \times \omega \times S}{4} = \frac{1}{4}\omega \times S \times (\frac{1}{\gamma_p} + \frac{1}{\Gamma_{ediff\_p}})^{-1} \quad \text{S11}$$

$$k_w = \frac{\gamma_{eff} \times \omega}{4} \times \frac{S_{OFR}}{V_{OFR}} = \frac{\omega}{D_{OFR\_In}} \times (\frac{1}{\gamma_w} + \frac{1}{\Gamma_{ediff\_w}})^{-1} \quad \text{S12}$$

Where k_g represents the gas-phase rate constant of reacting with BVOCs, ignoring the NO_3• reactions with oxidized organic products. k_p and k_w represent pseudo-first-order loss rate to the particle surface and to the inner reactor wall, respectively. γ_{eff} is the effective uptake coefficient for the gas G. ω is the molecular speed of gas G (ω, m s^{-1}). S is the total particle surface area (cm^2 m^{-3}). In this study, the output surface area of the particles was measured by the SMPS. We assume that the particle surface area was linearly correlated with the consumed BVOCs in the OFR_{NO_3}. S_{AFR} and V_{AFR} are the inner surface area and volume of the reactor. D_{OFR_In} is the inner diameter of the flow tube reactor. γ_p and γ_w are uptake coefficient to the particle surface and to OFR_{NO_3} inner wall. Γ_{diff} is the gas phase diffusion limitation in particles and reactor surfaces. For the uptake onto monodisperse spherical particles, $\Gamma_{diff\text{-}p}$ is generally described by the Fuchs-Sutugin equation in S13:[11,12]

$$\frac{1}{\Gamma_{ediff\_p}} = \frac{0.75 + 0.286 \times K_n}{K_n \times (K_n + 1)} \quad \text{S13}$$

Where Kn is Knudsen number can be calculated from the gas-phase diffusion coefficient (D, torr cm^2 s^{-1}), the molecular speed (ω), and particle diameter (D_p).

$$K_n = \frac{6D}{\omega \times D_p} \quad \text{S14}$$

When the loss rate of gas to the wall is not determined by surface reactivity, but by the diffusion through the gas-phase ($\gamma_w > \Gamma_{wall}$ ~7×10^{-6}), the following expression holds:

$$k_w = \frac{\omega}{D_{OFR\_In}} \times (\frac{1}{\gamma_p} + \frac{1}{\Gamma_{ediff\_w}})^{-1} \approx \frac{\omega \times \Gamma_{ediff_w}}{D_{OFR_{In}}} = \frac{4 \times 3.66 \times D}{{D_{OFR\_In}}^2} \quad \text{S15}$$

Equation S12 is valid for Peclet numbers (Pe = Dint × ν / D, ν is the velocity of the gas flow) excess ~20.[13] The Peclet numbers were estimated ~37 for N_2O_5 and ~26 for NO_3• in this study.

The reactive uptake coefficient (γ) depends on the reactant and particle surface available.[14] For NO_3•, the uptake coefficients by organics vary considerably from ~1×10^{-3} on saturated alkanes, alcohols, and acids, and up to $\gamma \approx 0.1$ for SOA contains double bonds.[8] In this study, the generated $BSOA_{NO_3}$ contain a lot of unsaturated products; thus, γ for NO_3• was assumed to be 0.1. Previous study has investigated the reactive uptake of N_2O_5 by alkenoic acid, alkanoate, and polyalcohol substrates. The reactive uptake coefficient varies from none to 8.14×10^{-4}. Thus a value of 4×10^{-4} was used for γ of N_2O_5 in this study. The averaged gas-phase diffusion coefficient of NO_3• is 92 ± 46 torr cm^2 s^{-1}, and N_2O_5 has a diffusion coefficient of 65 ± 33 torr cm^2 s^{-1}.[15] Here, based on number size distributions of the produced particles from studied BVOCs, the integrated heterogeneous reaction rates (1 atm and 295K) and wall loss rates for NO_3• and N_2O_5 were calculated and listed in Table S2:

Text 4.2 Box model simulation

A simplified model including reactions listed in Tables S3 was developed in a complex pathway simulator (COPASI, http://copasi.org/) to estimate the fate of gas species in the OFR_{NO_3}. Figure S6 presents the time traces of BVOCs, NO_3•,

and N_2O_5 in the OFR. It is noteworthy that the rapid conversion of N_2O_5 to NO_3• occurred in the OFR_{NO_3}. Although the wall loss of N_2O_5 dominated in the N_2O_5 loss (34-84%), N_2O_5 loss through thermal dissociation to NO_3• contributed significantly (13-64 %) as shown in Table S4. For experiments run at N_2O_5/VOC ratio below 3, the amount of NO_3 or N_2O_5 taken up by the particles ($F_{NO_3\_Par}$, $F_{N_2O_5\_Par}$) was negligible compared to the consumption of NO_3• by VOC. However, as the N_2O_5/VOC exceeded 3, the NO_3• and N_2O_5 uptake by particles became considerable, which indicates the importance of heterogeneous reaction in the reactor. The modeling revealed that the reactive uptake of NO_3• by the SOA particles was about an order magnitude higher than that of N_2O_5, highlighting the importance of NO_3• oxidation pathway in the OFR.

Text S5. Offline chemical composition analysis of $BSOA_{NO_3}$.

Upon extraction, 100 μL camphor sulfonic acid (Sigma Aldrich, 99 % purity) solution (10 mg L^{-1} in acetonitrile, Fisher Optima, LC-MS grade) and 100 μL caffeine (Sigma Aldrich, ReagentPlus) solution (10 mg L^{-1} in acetonitrile, Fisher Optima, LC-MS grade) are spiked onto the filters and used as internal standards. The filters were extracted twice with 6 mL acetonitrile (3 mL + 3 mL) using a voltex for 15 minutes. The two fractions were combined and then evaporated to 200 μL by using a gentle N_2 flow. Finally, the concentrated solutions were analyzed with ultra-high-performance liquid chromatography (HPLC) equipped with photodiode array (PDA) detector (spectra detection range of 200−800 nm) followed by Q-Exactive Hybrid Quadrupole-Orbitrap mass spectrometer (Orbitrap MS) with a standard heated electrospray ionization (HESI) source. The separation was performed using a Waters Acquity HSS T3 column (1.8 μm, 100 × 2.1 mm) with mobile phases consisting of (A) 0.1% formic acid (Fisher Optima, LC-MS grade) in water (Fisher Optima, LC-MS grade) and (B) 0.1% formic acid in acetonitrile. Gradient elution was performed by the A/B mixture at a total flow rate of 300 μL/min: 1% B for 2 min, a linear gradient to 100% B in the next 11 min, 100% B for another 2 min, back to 1% B in 0.1 min, and then kept for 6.9 min. The HESI settings were as follows: 4.0 kV spray potential, 35 units of sheath gas flow, 10 units of auxiliary gas flow, and 8 units of sweep gas flow. The capillary temperature was optimized to 250 °C to minimize the thermal decomposition of ON. Separately, online HESI/HRMS data were acquired in both positive and negative ion modes. The HESI was operated using a spray voltage of −2.6 and 3.2 kV for (−) and (+) modes, respectively. The mass resolving power was 140,000 at m/z 200, and the scanning range was set to m/z 50–750. The mass spectrometer was externally mass calibrated daily using a 2 mM sodium acetate solution (Sigma Aldrich, 99 %) that provided a series of negative and positive adduct ions in the range of m/z 50–750. The Xcalibur (Thermo Scientific) software was used to acquire the raw data and analysis. Moreover, the HRMS data was also processed with an open-source software toolbox, MZmine 2.39 (http://mzmine.github.io/), to perform peak deconvolution and chromatogram construction. Formula assignments were completed using the following constraints: C≤50, H≤100, N≤4, O≤50). Procedures and settings of the MZmine 2.39 are provided as below.

1. Masses from each scan were determined with noise cutoffs of 500 (HESI−) and 5000 (HESI+) followed by the application of a Fourier Transform Mass Spectrometer shoulder peak detection with a mass resolution of 140000 at *m/z* 200.
2. Automated Data Analysis Pipeline (ADAP) chromatogram builder[16] was chosen to rebuild chromatograms using a minimum group size of 6 and an intensity threshold of 100. Mass tolerances are fixed to 3 ppm (for the HESI−) and 5 ppm (for the HESI+) in the chromatogram builder.
3. Peaks were smoothed with a filter width of 5 and then deconvoluted by the ADAP Module Disclaimer (S/N threshold

5; minimum feature height of 1000; peak duration ranges of 0.07-2 min and retention time (RT) wavelet range of 0−0.1 min.

4. Isotope formulas were removed using the Collection of Algorithms for MEtabolite pRofile Annotation (CAMERA) search (m/z tolerance of 5 ppm; correlation threshold of 0.75; correlation p-value of 0.05; maximum charge of 2).

5. Adducts were identified ($[M-H+ACN]^-$, 41.0266 *m/z*; $[M-H+CH_2O_2]^-$, 46.0055 *m/z*; $[M+Na]^+$, 21.9825 *m/z*; $[M+K]^+$, 37.9559 *m/z*; $[M+NH_4]^+$, 17.0265 *m/z*; $[M+H+ACN]^+$, 41.0266 *m/z*; $[M+H+CH_2O_2]^+$, 46.0055 *m/z*; $[M+H+CHO_2Na]^+$, 67.9874 *m/z*; RT tolerance of 0.1 min; *m/z* tolerance of 3 ppm for HESI- and 5 ppm for HESI+; max adduct peak height of 50%).

6. Identified peak lists were aligned with *m/z* tolerance of 3 ppm for HESI− and 5 ppm for HESI+. The absolute RT tolerance, weight for RT, and weight for *m/z* were 0.3 min, 2 and 3, respectively. To predict the formulae, we used exact masses of $[M+H]^+$, $[M+Na]^+$, $[M+NH_4]^+$, and $[M+K]^+$ with an *m/z* tolerance of 5 ppm and $[M-H]^-$ ions with an *m/z* tolerance of 3 ppm, respectively.

7. The formulae assignment were tentatively determined and constrained by a series of limits including: Elements ($C_{1-50}H_{0-100}O_{0-50}N_{0-4}$), element court heuristics, ($1.3 \leq H/C \leq 2.5$, $N/C \leq 0.5$, $O/C \leq 2$, $O/N \geq 3$; if NOPS all > 1 then N< 10, O < 20, P < 4, S < 3; if NOP all > 3 then N < 11, O < 22, P < 6; if OPS all > 1 then O < 14, P < 3, S < 3; if PSN all > 1 then P < 3, S < 3, N < 4; if NOS all > 6 then N < 19, O < 14, S < 8), RDBE (0−15), and isotope pattern filter (isotope *m/z* tolerance of 5 ppm, minimum absolute intensity of 100).

Supporting Figures

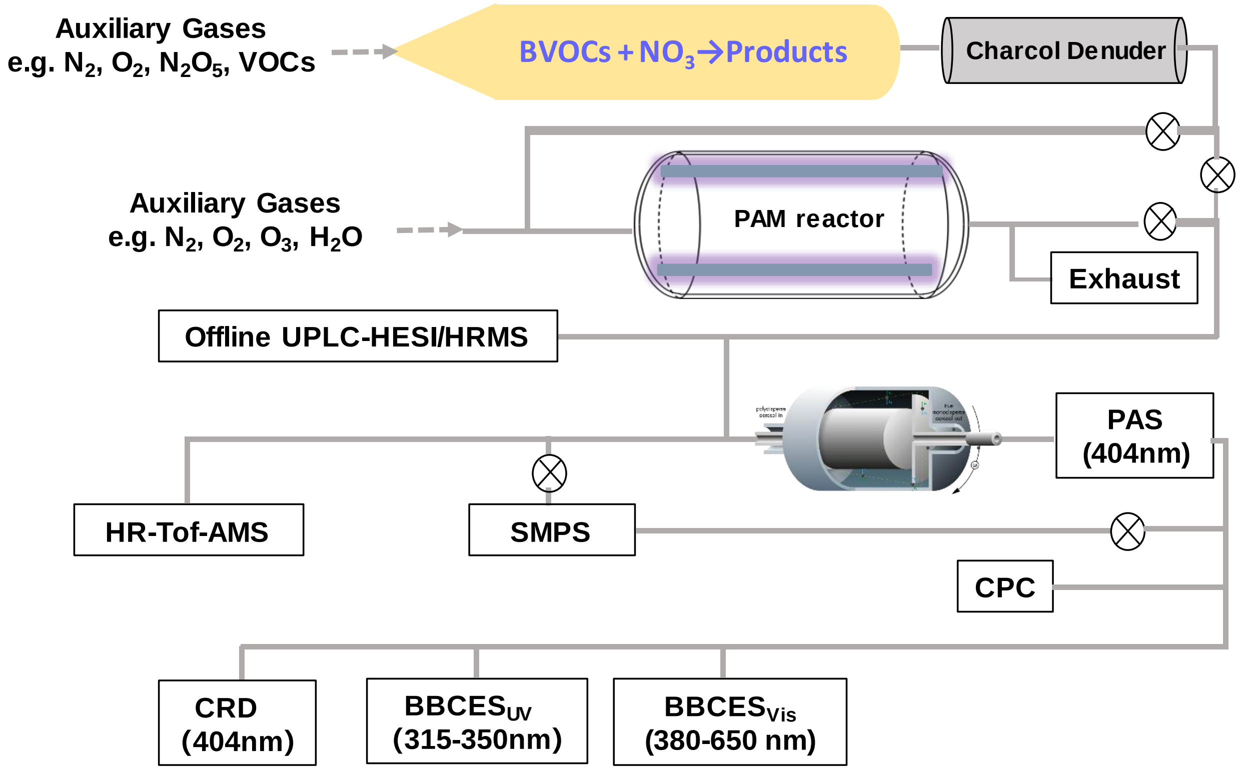

Figure S1. Experimental setup. Secondary organic aerosols (SOA) were generated in a glass oxidation flow reactor (OFR). A charcoal denuder removed VOCs and NOx immediately after the OFR. The particles were size−selected using an aerodynamic aerosol classifier (AAC) and then directed into a photoacoustic absorption spectrometer (PAS) for absorption measurement. The aerosol flow emerging the PAS was split for particle size distribution, total number concentration, and light extinction measurement. Two instruments were used for extinction measurement, a cavity ring-down spectroscopy (CRD) and a broadband cavity-enhanced spectrometer, which consists of a UV channel ($BBCES_{UV}$, 315-350 nm) and a visible change ($BBCES_{vis}$, 380-700 nm). Meanwhile, part of the aerosol was sampled by a high-resolution time-of-flight mass spectrometer (HR-Tof-AMS) for bulk chemical composition analysis. Filters were collected and used for offline analysis by high-performance liquid chromatography coupled with a photodiode array detector and a high-resolution Orbitrap mass spectrometer (HPLC-PDA-HESI/HRMS).

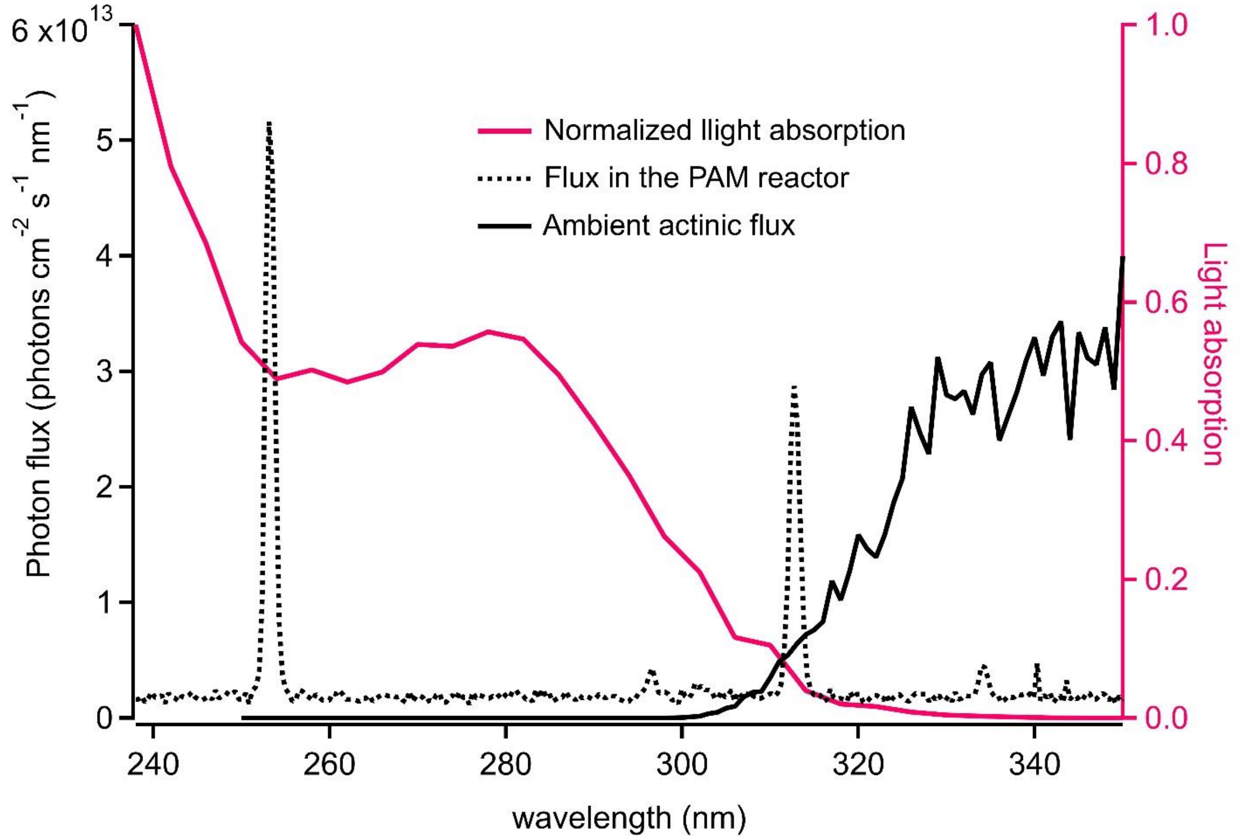

Figure S2. Determination of the equivalent photolysis time in the PAM. Left axis indicates the photon flux in the PAM reactor and the daily averaged actinic flux in Rehovot, Israel on December 17th, 2019, using the Tropospheric Ultraviolet and Visible (TUV) model from NCAR. The absorption spectra (red trace) at elution time of 11.1 min from the α-humulene derived $BSOA_{NO_3}$ was normalized to the highest absorption in the wavelength range of 250-350 nm.

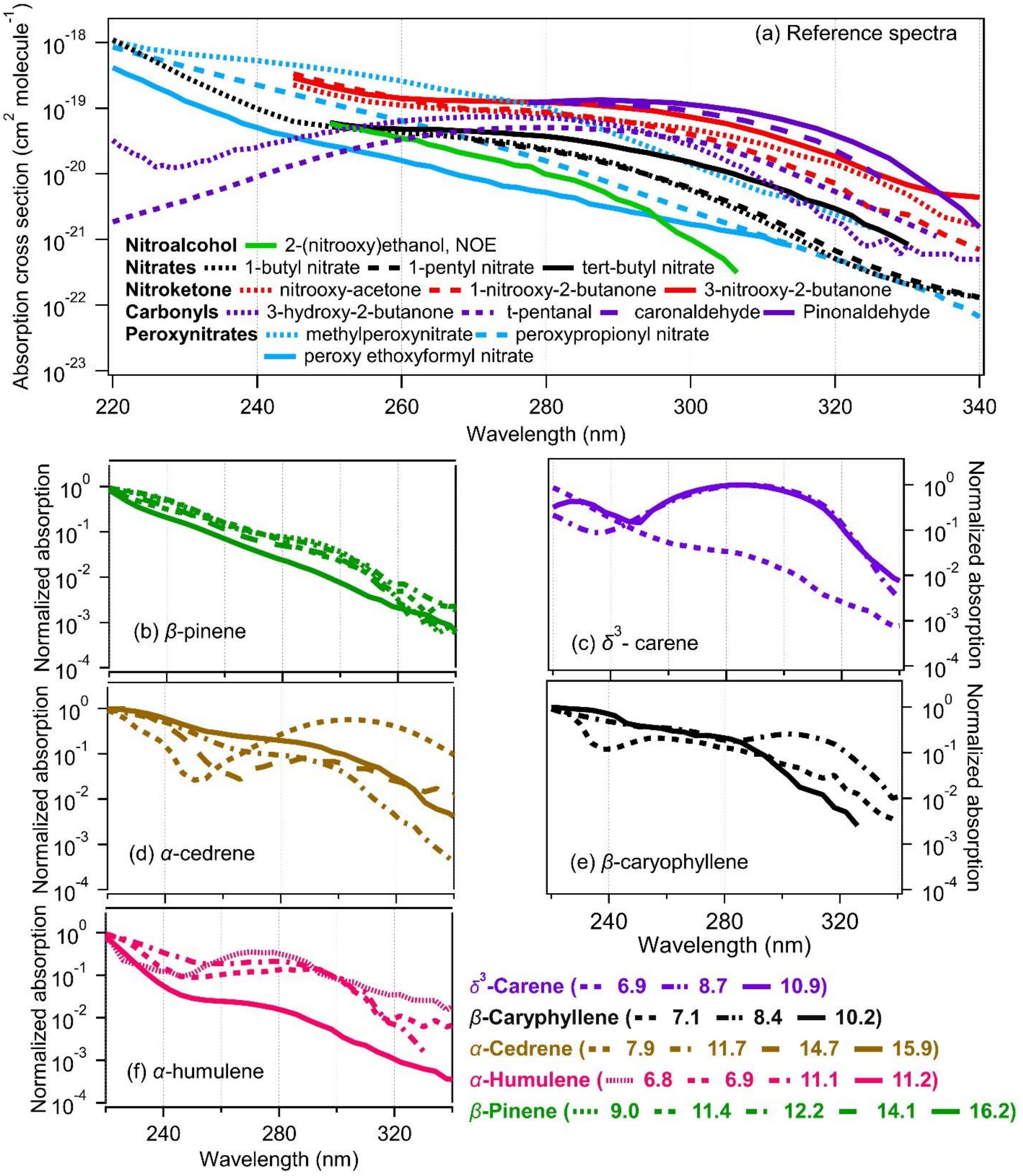

Figure S3. Absorption spectra of various reference compounds and compounds of interest in the $BSOA_{NO_3}$. Panel (a) shows the absorption cross section of different types of organic nitrates and carbonyls measured by previous studies.[17-24] Panels (b-f) display the absorption spectra measured by PDA in this study. Numbers in brackets are LC retention time. The absorption spectra are normalized to the highest absorption in the wavelength range of 220-340 nm.

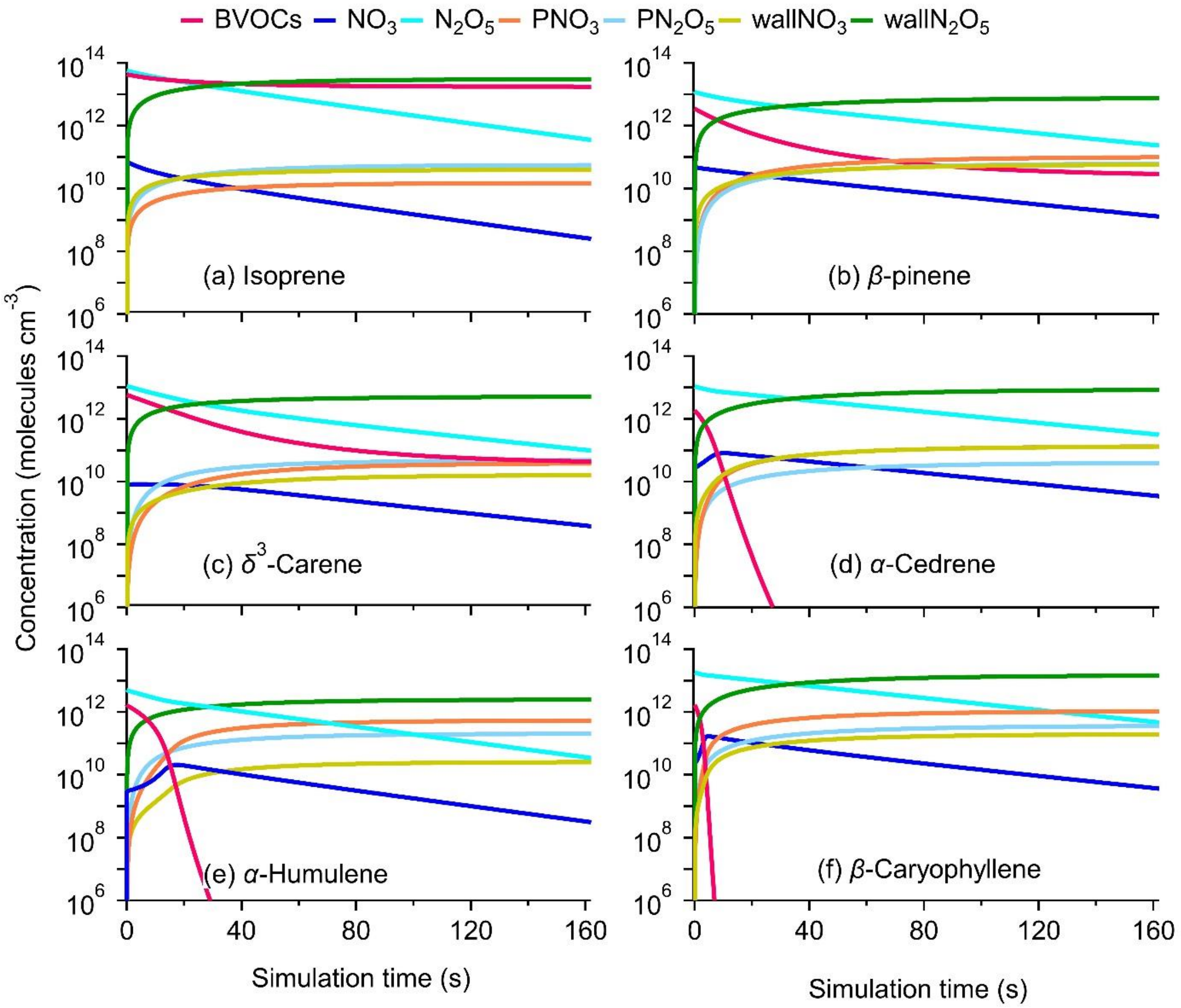

Figure S4. Box model simulated fates of N_2O_5, NO_3•, and BVOCs. Panel b and panel e show the simulation performed for β-pinene and α-humulene under the N_2O_5/VOC ratio of 3.4 and 3.0, respectively.

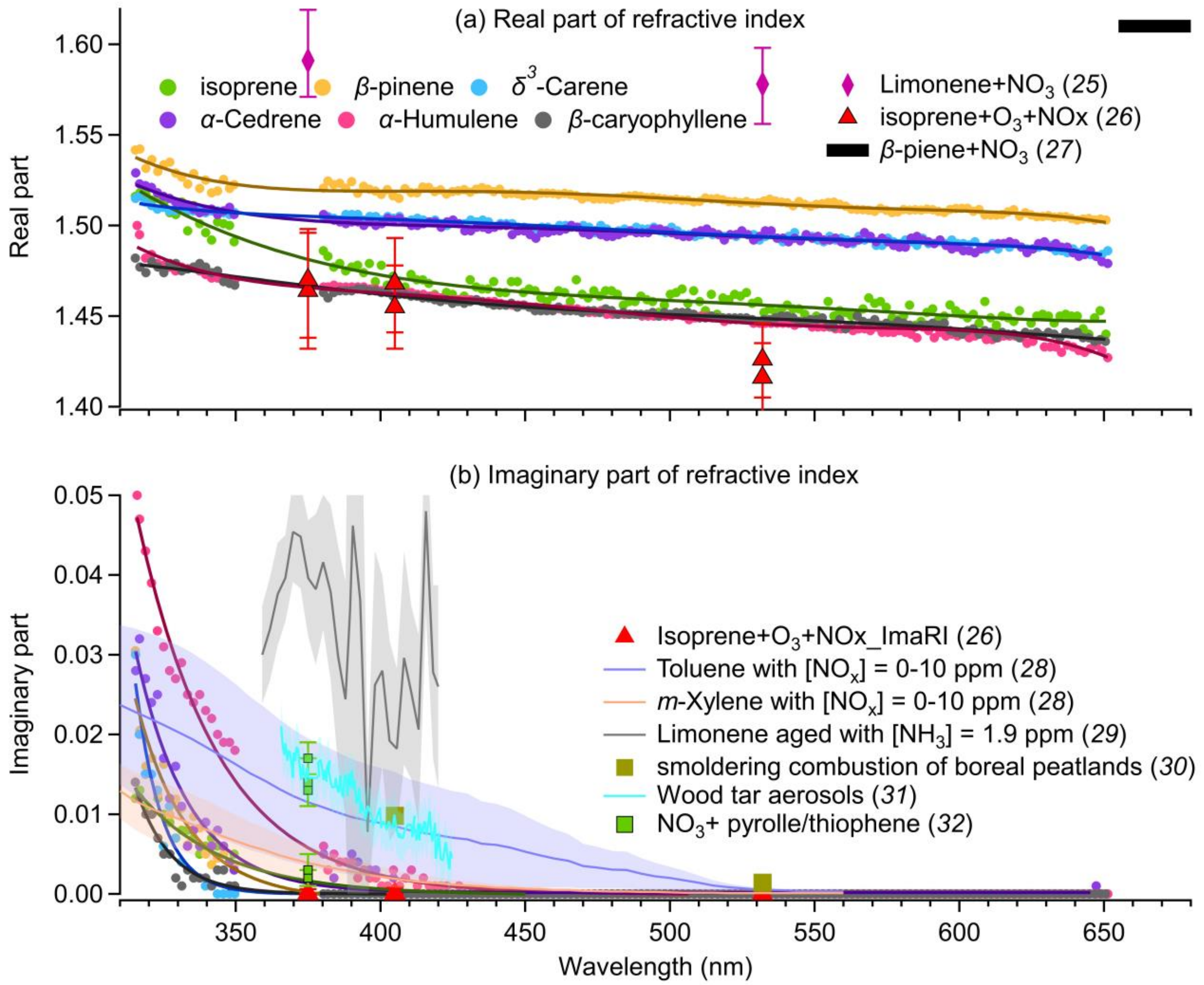

Figure S5. The refractive index for $BSOA_{NO_3}$ generated in this study and literature results. For literature results, the real RI are only summarized for SOA generated by NO_3• oxidation,[25-27] while for imaginary RI, data for SOA from anthropogenic VOCs[28] and BVOCs[26,29], biomass burning aerosols[30,31] and SOA from unsaturated heterocyclic VOCs[32] are shown.

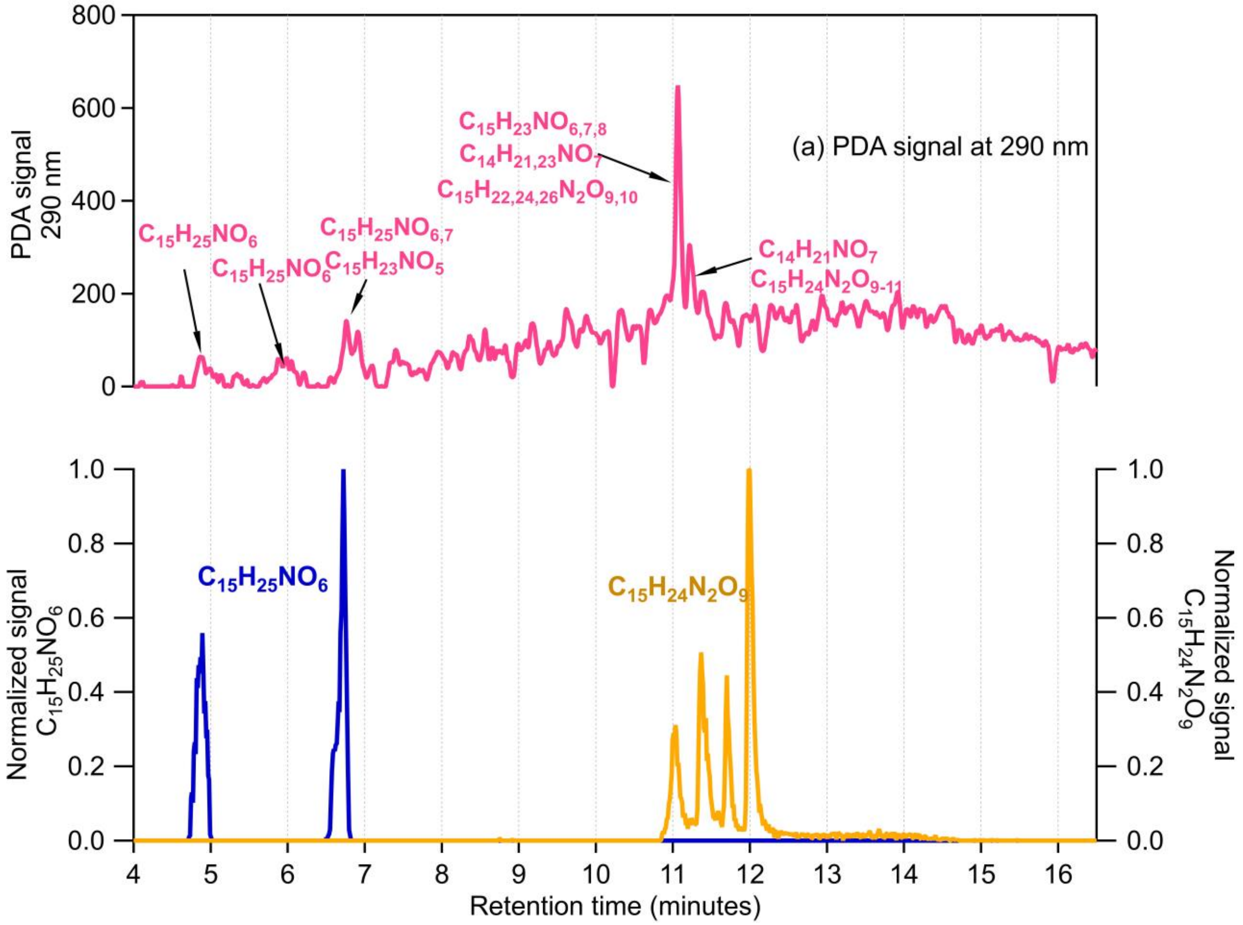

Figure S6. The absorption and extracted ion chromatograms for $BSOA_{NO_3}$ generated from the α-humulene. There are two $C_{15}H_{25}NO_6$ isomers and four isomers with the formula of $C_{15}H_{24}N_2O_9$.

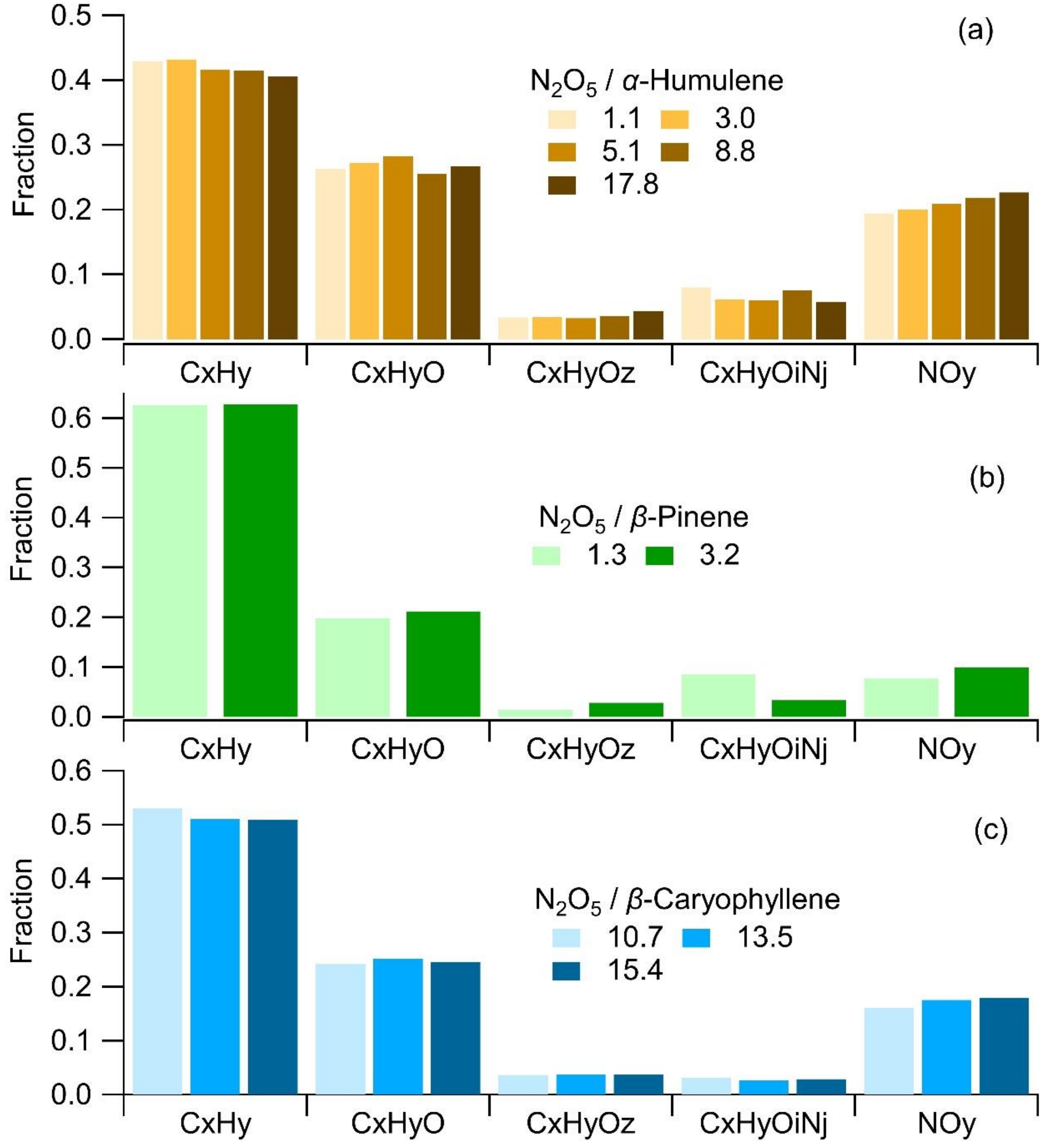

Figure S7. Influence of N_2O_5/VOC ratio on the fragments composition of SOA by NO_3• oxidation of *β*-pinene (a), *β*-caryophyllene (b), and *α*-humulene (c).

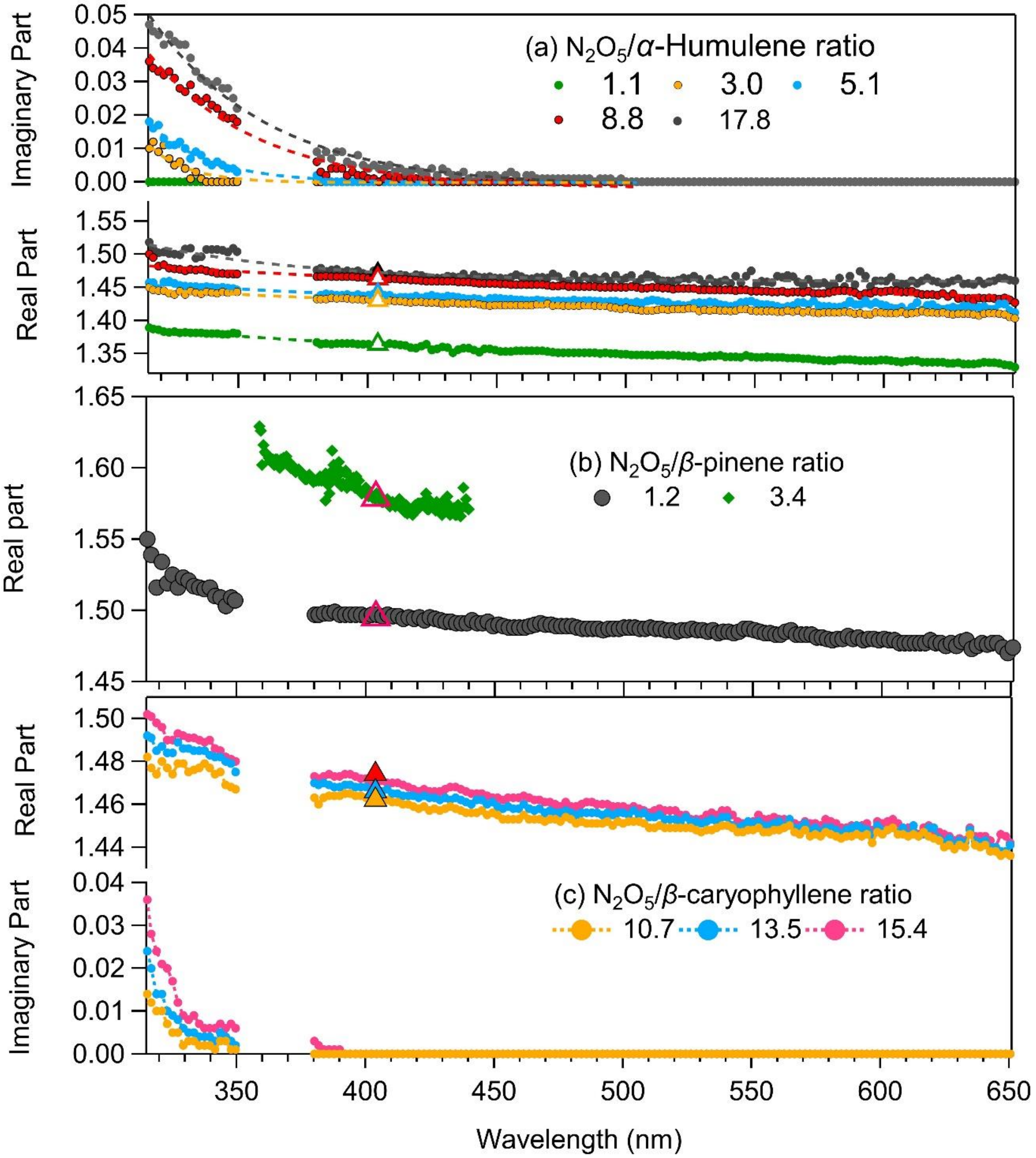

Figure S8. Influence of the N_2O_5/VOC ratio on the complex refractive index of the SOA from NO_3• oxidation. Panels (a)-(c) shown the data for α-humulene, β-pinene, and β-caryophyllene, respectively. Round markers represent results from BBCES while triangles display results from CRD. Both the real part and imaginary part of RI increase with the increasing ratio of N_2O_5/VOC.

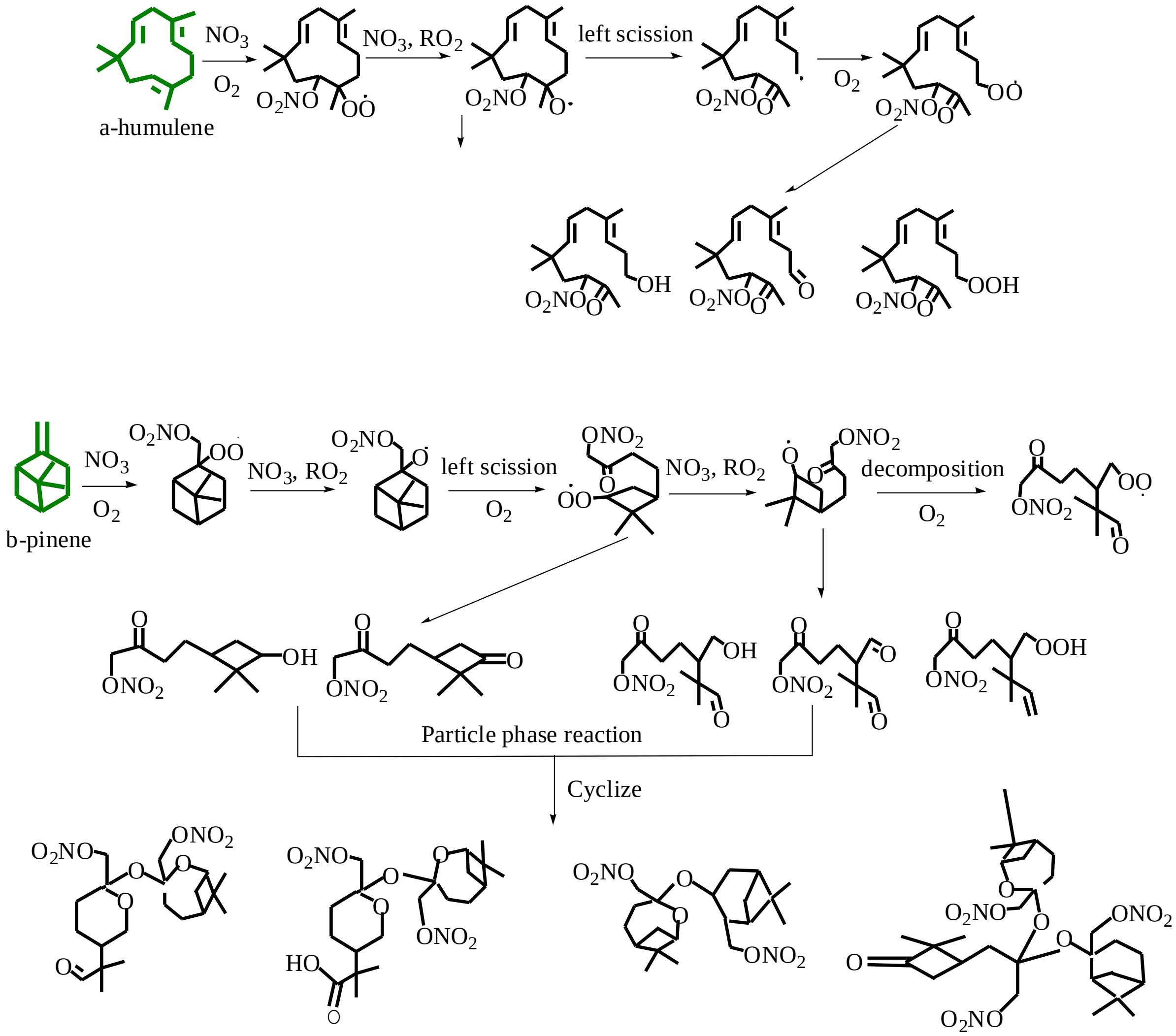

Figure S9. The NO3• initialed oxidation mechanism of *β*-pinene and *α*-humulene. The reactions mechanisms for *β*-pinene were adopted from the study by Claflin and Ziemann,[33] while those for *α*-humulene were proposed according to the result by Draper et al..[34]

Supporting Tables

Table S1. SOA generation and chemical-physical characteristics of the produced $BSOA_{NO_3}$.

BVOCs	mixing ratio (ppb)	N_2O_5/VOC ratio	N/C	H/C	O/C	OM/OC	f_{NO3}	f_{43}	f_{44}	NO^+ / NO_2^+	Density g/cm^3
Isoprene	1700	1.3	0.111	1.631	0.335	1.713	0.338	0.186	0.029	6.5	1.36
β-Pinene	41	1.2	0.044	1.610	0.113	1.337	0.154	0.041	0.006	5.4	1.19
β-Pinene	136	3.4	0.010	1.615	0.132	1.359	0.168	0.042	0.010	6.4	1.37
δ^3-Carene	227	1.9	0.007	1.564	0.328	1.577	0.212	0.247	0.027	5.3	1.23
α-Cedrene	73	5.8	0.009	1.563	0.122	1.304	0.110	0.096	0.012	9.5	1.18
α-Humulene	64	1.1	0.057	1.649	0.208	1.474	0.194	0.186	0.030	5.4	1.01
α-Humulene	64	3.0	0.043	1.649	0.214	1.473	0.201	0.186	0.030	5.3	1.17
α-Humulene	64	5.1	0.041	1.664	0.216	1.487	0.209	0.180	0.032	5.1	1.21
α-Humulene	64	8.8	0.054	1.669	0.217	1.493	0.219	0.182	0.034	4.9	1.23
α-Humulene	57	17.8	0.038	1.672	0.237	1.499	0.227	0.187	0.040	5.2	1.31
β-Caryophyllene	66	10.7	0.009	1.538	0.171	1.369	0.161	0.127	0.013	7.0	1.11
β-Caryophyllene	66	13.5	0.008	1.535	0.185	1.386	0.175	0.126	0.016	6.8	1.13
β-Caryophyllene	66	15.4	0.009	1.537	0.201	1.409	0.180	0.129	0.018	6.8	1.16

Table S2. Estimated reaction kinetics for NO_3• and N_2O_5 in the OFR_{NO_3}

VOCs	N_2O_5/VOC ratios	Maximum uptake rate (k_p, s^{-1})		Wall loss rate (k_w, s^{-1})	
		NO_3•	N_2O_5	NO_3•	N_2O_5
Isoprene	1.3	0.02	0.00005	0.03±0.02	0.02±0.01
δ^3-Carene	1.9	0.09	0.00029		
α-Cedrene	5.8	0.03	0.00010		
β-pinene	1.2	0.02	0.000066		
	3.4	0.07	0.00022		
α-humulene	1.1	0.66	0.0021		
	3	0.65	0.0020		
	8.8	0.65	0.0020		
	17.8	0.45	0.0014		
β-Caryophyllene	10.7	0.16	0.00051		
	13.5	0.14	0.00042		
	15.4	0.17	0.00052		

Table S3. List of reactions and their rate constants for the BVOCs+NO_3 simulation.

Reaction	Rate constant[a]
NO_2 + NO_3• = N_2O_5	$K_{eq} = 4.22 \times 10^{-11}$ [b]
NO + NO_3• → 2 NO_2	1.40×10^{-12} [b]
NO_3• + NO_2 → NO + NO_2 + O_2	6.30×10^{-19} [b]
NO_3• → wallNO_3[c]	0.03 s^{-1}
N_2O_5 → wallN_2O_5[c]	0.02s s^{-1}
isoprene + NO_3• → isoprene (P)	7.00×10^{-13}
N_2O_5 (Iso)→ PN_2O_5 (Iso)[d]	5×10^{-5} s^{-1}
NO_3• (Iso)→ PNO_3 (Iso)[d]	0.02 s^{-1}
β-pinene + NO_3• → *β*-pinene (P)	2.51×10^{-12}
N_2O_5 (Pin)→ PN_2O_5 (Pin)	6.6/22 × 10^{-5} s^{-1}
NO_3• (Pin)→ PNO_3 (Pin)	0.021/0.065 s^{-1}
δ[3]-carene + NO_3• → *δ*[3]-carene (P)	9.10×10^{-12}
N_2O_5 (Car)→ PN_2O_5 (Car)	2.9×10^{-4} s^{-1}
NO_3• (Car)→ PNO_3 (Car)	0.09 s^{-1}
α-cedrene + NO_3• → *α*-cedrene (P)	8.20×10^{-12}
N_2O_5 (Ced)→ PN_2O_5 (Ced)	1.0×10^{-4} s^{-1}
NO_3• (Ced)→ PNO_3 (Ced)	0.03 s^{-1}
α-humulene + NO_3• → *α*-humulene (P)	3.90×10^{-11}
N_2O_5 (Hum)→ PN_2O_5 (Hum)	2.1/2.0/2.0/1.4 × 10^{-3} s^{-1}
NO_3• (Hum)→ PNO_3 (Hum)	0.66/0.65/0.65/0.45 s^{-1}
β-caryophyllene + NO_3• → *β*-caryophyllene (P)	1.90×10^{-11}
N_2O_5 (Cary)→ PN_2O_5 (Cary)	5.1/4.2/5.2 × 10^{-4} s^{-1}
NO_3• (Cary)→ PNO_3 (Cary)	0.16/0.14/0.17 s^{-1}

[a] Unless noted, reaction rate constants are extracted from as referred to that of *β*-caryophyllene and *α*-pinene in MCM v3.3. The unit of the rate constants are cm^3 $molecules^{-1}$ s^{-1}.
[b] Reaction rates are adopted from JPL kinetics and references therein [35].
[c] P represents the uptake by particles.
[d] wall represents the wall loss pathway.

Table S4. Loss of NO_3• and N_2O_5 in the OFR_{NO_3}.

VOCs	N_2O_5/VOC	$F_{NO_3\_VOCs}$	$F_{NO_3\_Par}$	$F_{NO_3\_wall}$	$F_{N_2O_5\_Par}$	$F_{N_2O_5\_wall}$	$F_{N_2O_5\_NO_3}$
Isoprene	1.3	0.998	0.001	0.002	0.001	0.534	0.460
δ^3-Carene	1.9	0.990	0.007	0.003	0.004	0.466	0.523
α-Cedrene	5.8	0.873	0.063	0.064	0.004	0.794	0.195
β-pinene	1.2	0.968	0.014	0.018	0.002	0.665	0.311
	3.4	0.959	0.026	0.015	0.005	0.631	0.320
α-humulene	1.1	0.987	0.013	0.001	0.023	0.336	0.641
	3	0.748	0.240	0.011	0.041	0.502	0.450
	8.8	0.440	0.536	0.025	0.063	0.656	0.268
	17.8	0.341	0.617	0.041	0.052	0.763	0.182
β-Caryophyllene	10.7	0.565	0.367	0.069	0.020	0.813	0.159
	13.5	0.537	0.378	0.085	0.017	0.843	0.131
	15.4	0.477	0.442	0.081	0.021	0.842	0.128

Note: $F_{NO_3\_VOCs}$, $F_{NO_3\_Par}$, and $F_{NO_3\_wall}$ represent the contribution of NO_3• loss due to reaction with VOCs, uptake by particles, and wall loss, respectively. $F_{N_2O_5\_Par}$, $F_{N_2O_5\_wall}$, and $F_{N_2O_5\_NO_3}$ represents the faction of N_2O_5 loss due to uptake by particles, wall loss, and conversion to NO_3•, respectively.

Table S5. Refractive index measured for different types of $BSOA_{NO_3}$ in this study.

Compounds	N_2O_5/VOC ratio	Real Part				Imaginary Part	
		404 nm (CRD)	316 nm	330 nm	532 nm	316 nm	330 nm
Isoprene	1.3	1.481 ± 0.002	1.517 ± 0.004	1.505 ± 0.003	1.455 ± 0.005	0.011 ± 0.009	0.008 ± 0.011
β-Pinene	1.2	1.495 ± 0.001	1.545 ± 0.001	1.522 ± 0.001	1.486 ± 0.001	0.003 ± 0.005	0.001 ± 0.002
β-Pinene	3.4	1.579 ± 0.001					
δ^3-Carene	1.9	1.501 ± 0.001	1.511 ± 0.006	1.509 ± 0.002	1.493 ± 0.001	0.031 ± 0.007	0.006 ± 0.003
α-Cedrene	5.8	1.488 ± 0.001	1.526 ± 0.003	1.489 ± 0.025	1.496 ± 0.003	0.03 ± 0.012	0.013 ± 0.016
α-Humulene	1.1	1.364 ± 0.001	1.388 ± 0.004	1.382 ± 0.001	1.346 ± 0.001		
α-Humulene	3.0	1.430± 0.001	1.448 ± 0.007	1.442 ± 0.002	1.416 ± 0.002	0.011 ± 0.005	0.003 ± 0.004
α-Humulene	5.1	1.439 ± 0.001	1.457 ± 0.008	1.452 ± 0.003	1.427 ± 0.001	0.017 ± 0.004	0.009 ± 0.003
α-Humulene	8.8	1.463 ± 0.001	1.498 ± 0.004	1.476 ± 0.002	1.446 ± 0.001	0.035 ± 0.005	0.028 ± 0.003
α-Humulene	17.8	1.470 ± 0.001	1.513 ± 0.007	1.509 ± 0.005	1.458 ± 0.004	0.046 ± 0.009	0.039 ± 0.008
β-Caryophyllene	10.7	1.462 ± 0.001	1.48 ± 0.006	1.477 ± 0.002	1.448 ± 0.001	0.013 ± 0.002	0.002 ± 0.003
β-Caryophyllene	13.5	1.466 ± 0.001	1.492 ± 0.007	1.486 ± 0.002	1.452 ± 0.001	0.022 ± 0.009	0.004 ± 0.001
β-Caryophyllene	15.4	1.474 ± 0.001	1.502 ± 0.007	1.492 ± 0.003	1.455 ± 0.001	0.032 ± 0.012	0.009 ± 0.002

Table S6. The effect of OH• aging and photolysis on the chemical composition of the produced $BSOA_{NO_3}$.

System investigated	f_{NO_3}	N/C	H/C	O/C
β-pinene+NO_3	0.201	0.074	1.560	0.183
β-pinene+NO_3+OH aging	0.196	0.072	1.547	0.189
β-pinene+NO_3+Photolysis	0.195	0.071	1.541	0.188
α-humulene+NO_3	0.206	0.084	1.609	0.251
α-humulene+NO_3+OH aging	0.192	0.078	1.614	0.273
α-humulene+NO_3+Photolysis	0.191	0.075	1.613	0.244

Supporting References

(1) Suarez-Bertoa, R.; Picquet-Varrault, B.; Tamas, W.; Pangui, E.; Doussin, J. F., Atmospheric Fate of a Series of Carbonyl Nitrates: Photolysis Frequencies and OH-Oxidation Rate Constants. *Environ. Sci. Technol.* **2012,** *46*, (22), 12502-12509.

(2) Müller, J. F.; Peeters, J.; Stavrakou, T., Fast photolysis of carbonyl nitrates from isoprene. *Atmos. Chem. Phys.* **2014,** *14*, (5), 2497-2508.

(3) Li, R.; Palm, B. B.; Ortega, A. M.; Hlywiak, J.; Hu, W.; Peng, Z.; Day, D. A.; Knote, C.; Brune, W. H.; de Gouw, J. A.; Jimenez, J. L., Modeling the Radical Chemistry in an Oxidation Flow Reactor: Radical Formation and Recycling, Sensitivities, and the OH Exposure Estimation Equation. *The Journal of Physical Chemistry A* **2015,** *119*, (19), 4418-4432.

(4) Peng, Z.; Day, D. A.; Stark, H.; Li, R.; Lee-Taylor, J.; Palm, B. B.; Brune, W. H.; Jimenez, J. L., HOx radical chemistry in oxidation flow reactors with low-pressure mercury lamps systematically examined by modeling. *Atmos. Meas. Tech.* **2015,** *8*, (11), 4863-4890.

(5) Gross, S.; Iannone, R.; Xiao, S.; Bertram, A. K., Reactive uptake studies of NO3 and N2O5 on alkenoic acid, alkanoate, and polyalcohol substrates to probe nighttime aerosol chemistry. *Phys. Chem. Chem. Phys.* **2009,** *11*, (36), 7792-7803.

(6) Chang, W. L.; Bhave, P. V.; Brown, S. S.; Riemer, N.; Stutz, J.; Dabdub, D., Heterogeneous Atmospheric Chemistry, Ambient Measurements, and Model Calculations of N2O5: A Review. *Aerosol Sci. Tech.* **2011,** *45*, (6), 665-695.

(7) Moise, T.; Talukdar, R. K.; Frost, G. J.; Fox, R. W.; Rudich, Y., Reactive uptake of NO3 by liquid and frozen organics. *Journal of Geophysical Research: Atmospheres* **2002,** *107*, (D2), AAC 6-1-AAC 6-9.

(8) Ng, N. L.; Brown, S. S.; Archibald, A. T.; Atlas, E.; Cohen, R. C.; Crowley, J. N.; Day, D. A.; Donahue, N. M.; Fry, J. L.; Fuchs, H.; Griffin, R. J.; Guzman, M. I.; Herrmann, H.; Hodzic, A.; Iinuma, Y.; Jimenez, J. L.; Kiendler-Scharr, A.; Lee, B. H.; Luecken, D. J.; Mao, J. Q.; McLaren, R.; Mutzel, A.; Osthoff, H. D.; Ouyang, B.; Picquet-Varrault, B.; Platt, U.; Pye, H. O. T.; Rudich, Y.; Schwantes, R. H.; Shiraiwa, M.; Stutz, J.; Thornton, J. A.; Tilgner, A.; Williams, B. J.; Zaveri, R. A., Nitrate radicals and biogenic volatile organic compounds: oxidation, mechanisms, and organic aerosol. *Atmos. Chem. Phys.* **2017,** *17*, (3), 2103-2162.

(9) Crowley, J. N.; Ammann, M.; Cox, R. A.; Hynes, R. G.; Jenkin, M. E.; Mellouki, A.; Rossi, M. J.; Troe, J.; Wallington, T. J., Evaluated kinetic and photochemical data for atmospheric chemistry: Volume V – heterogeneous reactions on solid substrates. *Atmos. Chem. Phys.* **2010,** *10*, (18), 9059-9223.

(10) Davidovits, P.; Kolb, C. E.; Williams, L. R.; Jayne, J. T.; Worsnop, D. R., Mass Accommodation and Chemical Reactions at Gas−Liquid Interfaces. *Chem. Rev.* **2006,** *106*, (4), 1323-1354.

(11) Pöschl, U.; Rudich, Y.; Ammann, M., Kinetic model framework for aerosol and cloud surface chemistry and gas-particle interactions – Part 1: General equations, parameters, and terminology. *Atmos. Chem. Phys.* **2007,** *7*, (23), 5989-6023.

(12) Ammann, M.; Pöschl, U., Kinetic model framework for aerosol and cloud surface chemistry and gas-particle interactions – Part 2: Exemplary practical applications and numerical simulations. *Atmos. Chem. Phys.* **2007,** *7*, (23), 6025-6045.

(13) Zasypkin, A. Y.; Grigor'eva, V. M.; Korchak, V. N.; Gershenson, Y. M., A Formula for Summing of Kinetic Resistances for Mobile and Stationary Media: I. Cylindrical Reactor. *Kinet Catal+* **1997,** *38*, 772-781.

(14) Kolb, C. E.; Cox, R. A.; Abbatt, J. P. D.; Ammann, M.; Davis, E. J.; Donaldson, D. J.; Garrett, B. C.; George, C.; Griffiths, P. T.; Hanson, D. R.; Kulmala, M.; McFiggans, G.; Poschl, U.; Riipinen, I.; Rossi, M. J.; Rudich, Y.; Wagner, P. E.; Winkler, P. M.; Worsnop, D. R.; O'Dowd, C. D., An overview of current issues in the uptake of atmospheric trace gases by aerosols and clouds. *Atmos. Chem. Phys.* **2010,** *10*, (21), 10561-10605.

(15) Tang, M. J.; Cox, R. A.; Kalberer, M., Compilation and evaluation of gas phase diffusion coefficients of reactive trace gases in the atmosphere: volume 1. Inorganic compounds. *Atmos. Chem. Phys.* **2014,** *14*, (17), 9233-9247.

(16) Myers, O. D.; Sumner, S. J.; Li, S.; Barnes, S.; Du, X., One Step Forward for Reducing False Positive and False Negative Compound Identifications from Mass Spectrometry Metabolomics Data: New Algorithms for Constructing Extracted Ion Chromatograms and Detecting Chromatographic Peaks. *Anal. Chem.* **2017,** *89*, (17), 8696-8703.

(17) Bossolasco, A. G.; Malanca, F. E.; Arguello, G. A., Peroxy ethoxyformyl nitrate, CH3CH2OC(O)OONO2. Spectroscopic and thermal characterization. *J. Photoch. Photobio. A* **2011,** *221*, (1), 58-63.

(18) Roberts, J. M.; Fajer, R. W., UV absorption cross sections of organic nitrates of potential atmospheric importance and estimation of atmospheric lifetimes. *Environ. Sci. Technol.* **1989,** *23*, (8), 945-951.

(19) Barnes, I.; Becker, K. H.; Zhu, T., Near UV absorption spectra and photolysis products of difunctional organic nitrates: Possible importance as NOxreservoirs. *J Atmos Chem* **1993,** *17*, (4), 353-373.

(20) Clemitshaw, K. C.; Williams, J.; Rattigan, O. V.; Shallcross, D. E.; Law, K. S.; Anthony Cox, R., Gas-phase ultraviolet absorption cross-sections and atmospheric lifetimes of several C2 · C5 alkyl nitrates. *Journal of Photochemistry and Photobiology A: Chemistry* **1997,** *102*, (2-3), 117-126.

(21) Atkinson, R.; Baulch, D. L.; Cox, R. A.; Crowley, J. N.; Hampson, R. F.; Hynes, R. G.; Jenkin, M. E.; Rossi, M. J.; Troe, J., Evaluated kinetic and photochemical data for atmospheric chemistry: Volume II - gas phase reactions of organic species. *Atmos. Chem. Phys.* **2006,** *6*, (11), 3625-4055.

(22) Messaadia, L.; El Dib, G.; Ferhati, A.; Roth, E.; Chakir, A., Gas phase UV absorption cross-sections for a series of hydroxycarbonyls. *Chem. Phys. Lett.* **2012,** *529*, 16-22.

(23) Bossolasco, A. G.; Malanca, F. E.; Argüello, G. A., Peroxy ethoxyformyl nitrate, CH3CH2OC(O)OONO2. Spectroscopic and thermal characterization. *Journal of Photochemistry and Photobiology A: Chemistry* **2011,** *221*, (1), 58-63.

(24) Hallquist, M.; WÄngberg, I.; LjungstrÖm, E., Atmospheric Fate of Carbonyl Oxidation Products Originating from α-Pinene and Δ3-Carene: Determination of Rate of Reaction with OH and NO3 Radicals, UV Absorption Cross Sections, and Vapor Pressures. *Environ. Sci. Technol.* **1997,** *31*, (11), 3166-3172.

(25) Peng, C.; Wang, W.; Li, K.; Li, J.; Zhou, L.; Wang, L.; Ge, M., The Optical Properties of Limonene Secondary Organic Aerosols: The Role of NO3, OH, and O3 in the Oxidation Processes. *J. Geophys. Res.-Atmos.* **2018,** *123*, (6), 3292-3303.

(26) Nakayama, T.; Sato, K.; Imamura, T.; Matsumi, Y., Effect of Oxidation Process on Complex Refractive Index of Secondary Organic Aerosol Generated from Isoprene. *Environ. Sci. Technol.* **2018,** *52*, (5), 2566-2574.

(27) Varma, R. M.; Ball, S. M.; Brauers, T.; Dorn, H. P.; Heitmann, U.; Jones, R. L.; Platt, U.; Pöhler, D.; Ruth, A. A.; Shillings, A. J. L.; Thieser, J.; Wahner, A.; Venables, D. S., Light extinction by secondary organic aerosol: an intercomparison of three broadband cavity spectrometers. *Atmos. Meas. Tech.* **2013,** *6*, (11), 3115-3130.

(28) Liu, P. F.; Abdelmalki, N.; Hung, H. M.; Wang, Y.; Brune, W. H.; Martin, S. T., Ultraviolet and visible complex refractive indices of secondary organic material produced by photooxidation of the aromatic compounds toluene and m-xylene. *Atmos. Chem. Phys.* **2015,** *15*, (3), 1435-1446.

(29) Flores, J. M.; Washenfelder, R. A.; Adler, G.; Lee, H. J.; Segev, L.; Laskin, J.; Laskin, A.; Nizkorodov, S. A.; Brown, S. S.; Rudich, Y., Complex refractive indices in the near-ultraviolet spectral region of biogenic secondary organic aerosol aged with ammonia. *Phys. Chem. Chem. Phys.* **2014,** *16*, (22), 10629-10642.

(30) Sumlin, B. J.; Pandey, A.; Walker, M. J.; Pattison, R. S.; Williams, B. J.; Chakrabarty, R. K., Atmospheric Photooxidation Diminishes Light Absorption by Primary Brown Carbon Aerosol from Biomass Burning. *Environ. Sci. Technol. Lett.* **2017**.

(31) Li, C.; He, Q.; Schade, J.; Passig, J.; Zimmermann, R.; Meidan, D.; Laskin, A.; Rudich, Y., Dynamic changes in optical and chemical properties of tar ball aerosols by atmospheric photochemical aging. *Atmos. Chem. Phys.* **2019,** *19*, (1), 139-163.

(32) Jiang, H. H.; Frie, A. L.; Lavi, A.; Chen, J. Y.; Zhang, H. F.; Bahreini, R.; Lin, Y. H., Brown Carbon Formation from Nighttime Chemistry of Unsaturated Heterocyclic Volatile Organic Compounds. *Environ. Sci. Technol. Lett.* **2019,** *6*, (3), 184-190.

(33) Claflin, M. S.; Ziemann, P. J., Identification and Quantitation of Aerosol Products of the Reaction of β-Pinene with NO3 Radicals and Implications for Gas- and Particle-Phase Reaction Mechanisms. *J. Phys. Chem. A* **2018,** *122*, (14), 3640-3652.

(34) Draper, D. C.; Myllys, N.; Hyttinen, N.; Møller, K. H.; Kjaergaard, H. G.; Fry, J. L.; Smith, J. N.; Kurtén, T., Formation of Highly Oxidized Molecules from NO3 Radical Initiated Oxidation of Δ-3-Carene: A Mechanistic Study. *ACS Earth and Space Chem.* **2019,** *3*, (8), 1460-1470.

(35) Burkholder, J. B.; Sander, S. P.; Abbatt, J. P. D.; Barker, J. R.; Huie, R. E.; Kolb, C. E.; Kurylo, M. J.; Orkin, V. L.; Wilmouth, D. M.; Wine, P. H., Chemical Kinetics and Photochemical Data for Use in Atmospheric Studies, Evaluation No. 18. *Jet Propulsion Laboratory* **2015**.
